# Mammalian HEMK1 methylates glutamine residue of the GGQ motif of mitochondrial release factors

**DOI:** 10.1038/s41598-022-08061-y

**Published:** 2022-03-08

**Authors:** Qi Fang, Yusuke Kimura, Tadahiro Shimazu, Takehiro Suzuki, Ayumi Yamada, Naoshi Dohmae, Shintaro Iwasaki, Yoichi Shinkai

**Affiliations:** 1 Cellular Memory Laboratory, RIKEN Cluster for Pioneering Research, RIKEN, Wako, Saitama 351-0198 Japan; 2 Graduate School of Science and Engineering, Saitama University, Saitama, 338-8570 Japan; 3 RNA Systems Biochemistry Laboratory, RIKEN Cluster for Pioneering Research, Wako, Saitama 351-0198 Japan; 4 Department of Computational Biology and Medical Sciences, Graduate School of Frontier Sciences, The University of Tokyo, Kashiwa, Chiba 277-8561 Japan; 5 Biomolecular Characterization Unit, Technology Platform Division, RIKEN Center for Sustainable Resource Science, Wako, Saitama 351-0198 Japan

**Keywords:** Biochemistry, Cell biology, Genetics, Molecular biology

## Abstract

Despite limited reports on glutamine methylation, methylated glutamine is found to be highly conserved in a "GGQ" motif in both prokaryotes and eukaryotes. In bacteria, glutamine methylation of peptide chain release factors 1/2 (RF1/2) by the enzyme PrmC is essential for translational termination and transcript recycling. Two PrmC homologs, HEMK1 and HEMK2, are found in mammals. In contrast to those of HEMK2, the biochemical properties and biological significance of HEMK1 remain largely unknown. In this study, we demonstrated that HEMK1 is an active methyltransferase for the glutamine residue of the GGQ motif of all four putative mitochondrial release factors (mtRFs)—MTRF1, MTRF1L, MRPL58, and MTRFR. In *HEMK1-*deficient HeLa cells, GGQ motif glutamine methylation was absent in all the mtRFs. We examined cell growth and mitochondrial properties, but disruption of the *HEMK1* gene had no considerable impact on the overall cell growth, mtDNA copy number, mitochondrial membrane potential, and mitochondrial protein synthesis under regular culture condition with glucose as a carbon source. Furthermore, cell growth potential of *HEMK1* KO cells was still maintained in the respiratory condition with galactose medium. Our results suggest that HEMK1 mediates the GGQ methylation of all four mtRFs in human cells; however, this specific modification seems mostly dispensable in cell growth and mitochondrial protein homeostasis at least for HeLa cells under fermentative culture condition.

## Introduction

Post-translational modifications (PTMs) such as phosphorylation, acetylation, methylation, SUMOylation, can alter protein functions. Among these PTMs, research on protein methylation has attracted increasing attention due to its role in epigenetic regulations^1^. A large number of studies have been conducted on the methylation of lysine and arginine residues owing to their specific roles in histone PTMs, which are highly relevant in chromatin regulation and gene expression^2,3^. Besides adenosine triphosphate (ATP), *S*-adenosylmethionine (SAM)—the donor of methyl moiety for protein methylation—is the second most used cosubstrate in biological processes, suggesting that protein methylation is a pervasive biochemical event involved in different cellular processes^4,5^.

Glutamine methylation, although rarely studied, is one of the essential protein modifications in prokaryotes^6^. The Gly-Gly-Gln (GGQ) motif of release factors (RF1 and RF2) is a remarkable substrate for glutamine methylation. Generally, at the end of translation, the stop codons in transcripts are read by RF1 (UAA and UAG) and RF2 (UAA and UGA). Subsequently, these factors cleave the nascent polypeptide chain from the last linked tRNA in the peptidyl transferase center (PTC) of the ribosome. The GGQ motif in RF1 and RF2 plays a critical role in orienting a water molecule and facilitating a nucleophilic attack on the carbonyl carbon of the ester bond between the tRNA and the attached polypeptide. Glutamine methylation driven by the *N*^5^-glutamine methyltransferase (PrmC) at the GGQ motif in both bacterial RF1 and RF2 can stabilize the GGQ motif at the PTC through hydrophobic interactions, thus enhancing the polypeptide-releasing activity by about 5-fold and 20 fold, respectively^7–10^. Sequence and structural analyses revealed that PrmC is a typical seven-stranded β-sheet methyltransferase containing a GxGxG type SAM-binding motif and an Asn-Pro-Pro-Tyr (NPPY) glutamine/cystine-binding motif. Deletion of the SAM-binding motif of PrmC causes a loss of methylation in both RF1 and RF2 and results in an impaired growth phenotype in rich medium and lethality in nutrient-depleted medium^6^. In the *E. coli* genome, the *prmC* gene is positioned immediately downstream of its substrate (*prfA* gene, encoding RF1), ensuring in-time methylation of bacterial RF1^6^.

PrmC evolved divergently beyond the prokaryotic lineage. It evolved into a homolog protein Mtq2 in archaea (archaeal PrmC), with a slight difference in the molecular basis. A recent study published by van Tran et al*.*^11^ demonstrated that in *H. volcanii*, a PrmC homolog *Hvo*Mtq2 could methylate *Hvo*aRF1, with *Hov*aRF3 and GTP acting as cofactors. In addition, unlike the bacterial PrmC, *Hvo*Mtq2 forms a heterodimer with a protein called multifunctional methyltransferase subunit 112 (*Hvo*Trm112). Although this dimerization is not essential for the methyltransferase activity of *Hvo*Mtq2, the enzymatic activity of the *Hvo*Mtq2-*Hvo*Trm112 heterodimer is enhanced^11^. Similar to the phenotypes induced by *prmC* inactivation in bacteria, the deletion of the *HvoMtq2* gene also causes growth retardation in *H. volcanii*^11^.

In eukaryotic cells, two PrmC homologs, HEMK1 (also known as MTQ1 or MPRMC) and HEMK2 (also known as MTQ2 or N6AMT1), are localized in the mitochondria and cytosol, respectively^12,13^. HEMK2 exhibits similar enzymatic properties as those of the archaeal *Hvo*Mtq2. It requires the protein TRM112 as a binding partner and the eukaryotic peptide chain release factor subunit 3 (eRF3) and GTP as cofactors for efficient methylation of the eukaryotic peptide chain release factor subunit 1 (eRF1)^14^. The deletion of *scMtq2*, an HEMK2 homolog, in *S. cerevisiae* yielded a strain with multiple defects, including growth retardation, cold-sensitivity, and hypersensitivity to translational fidelity drugs^12^. It has been reported that the loss of HEMK2 leads to early embryonic lethality in mice^15^. In contrast to bacterial PrmC, mammalian HEMK2 has a broader range of substrate-binding activity, as it can also catalyze the methylation of lysine residues and nucleotides^16–18^.

Similarly, *sc*Mtq1, another PrmC homolog found in *S. cerevisiae*, can methylate one of the mitochondrial peptide chain release factors, Mrf1, required for translation termination inside the organelle^19^. The loss of methylation in Mrf1 shows no significant impact when solid media is used, in contrast to the *PrmC* knockout (KO) phenotype in prokaryotes. The growth rate is about 15% slower when non-fermentable carbon sources are used in liquid media, suggesting that the methylation in Mrf1 affects mitochondrial function and energy metabolism^12^. Considering the high sequence identity of the enzyme and substrates, it is possible that the mammalian HEMK1 also possesses a methyltransferase activity against human mitochondrial peptide chain release factors (mtRFs). To date, four potential mtRFs have been identified in humans, namely MTRF1, MTRF1L, MRPL58 (also known as ICT1), and MTRFR (also known as C12orf65)^20–23^. As with other class I release factors, all four mtRFs contain the universally conserved GGQ motif. Although direct evidence from LC–MS/MS is missing, previous studies have demonstrated that HEMK1 could cause a 14 Da increase in molecular mass in a GGQ-containing peptide fragment derived from MTRF1L, implying the addition of a methyl group on the glutamine residue of the GGQ motif^13^. Recently, Desai et al*.* demonstrated that MTRFR is methylated at position Q73 in its GGQ motif^24^. Glutamine methylation on MTRF1 and MTRL58 has so far been reported yet. Furthermore, it remains unclear how glutamine methylation of the GGQ motif affects the functions of mtRFs and mitochondrial translation. Thus, using proteomic analysis and genomic manipulation, we assessed the methyltransferase activity of HEMK1 towards the glutamine residue in GGQ motif of all potential mtRFs and examined possible biological consequences of HEMK1 depletion on mitochondrial properties.

## Results

### Domain and motif highlights of HEMK1 and mtRFs

**Figure 1:**
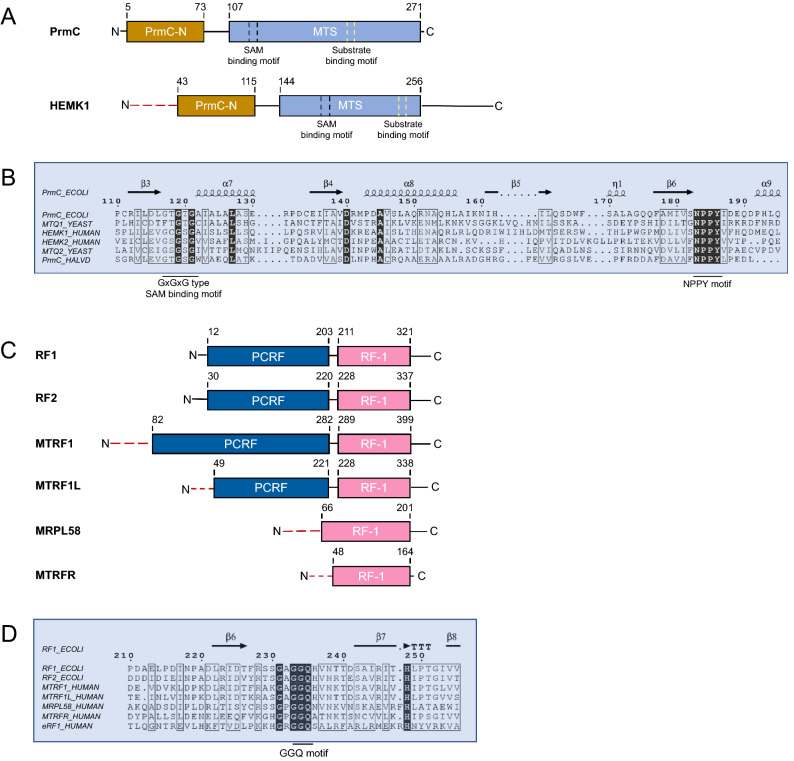
Domain architecture and sequence alignment of HEMK1 and mtRFs. (**A**) Domain architecture of PrmC and HEMK1. The red dash line indicated the predicted mitochondrial localization signal. PrmC-N: PrmC N-terminal domain (PF17827); MTS: methyltransferase small domain (PF05175). (**B**) Sequence alignments of various PrmC family proteins. The amino acid position is numbered according to the PrmC sequence. PCRF: peptide chain release factor domain, bacterial Class 1 (PF03462). RF-1: peptide chain release factor, bacterial Class I, PTH domain (PF00472). (**C**) Domain architecture of bacterial RF1, RF2, and mtRFs. The red dash lines indicated the predicted mitochondrial localization signal. (**D**) Sequence alignments of bacterial RF1, RF2, mtRFs, and the human eRF1. The amino acid position is numbered according to the bacterial RF1 sequence.

Human HEMK1 is a 338 amino acid residue protein that shows high sequence and structural similarity to bacterial PrmC (Fig. 1A,B, Figure S1). Similar to PrmC, HEMK1 belongs to the seven beta-strand class of methyltransferases. It possesses two distinct domains, a PrmC-N terminal domain (Pfam: PrmC_N, PF17827) and a methyltransferase small domain (Pfam: MTS, PF05175). The MTS domain contains a GxGxG type SAM-binding motif (position 117–121, referring to the bacterial PrmC sequence) and a NPPY glutamine/cystine-binding motif (position 183–186) (Fig. 1B, Figure S1). Sequence-based algorithms, such as DeepMito, MitoFate, and DISOPRED, predicted that the first 40 residues of the HEMK1 N-terminal constitute a disordered, amphiphilic region containing a mitochondrial localization signal (MLS) (Figure S2A; Table S1), suggesting its localization in the mitochondria.

Amino acid sequence comparison of HEMK1 with another human Mtq2 homolog, HEMK2, revealed a radical amino acid replacement at the − 1 position of the NPPY motif towards the N terminus. At this position, a large and hydrophobic residue, phenylalanine, was found in HEMK2, yeast Mtq2, and *Hvo*PrmC (*Hvo*Mtq2), but a small residue, serine/glycine, was found in HEMK1, yeast Mtq1, and PrmC (Fig. 1B). Considering the critical role of the NPPY motif in the PrmC methyltransferase family^14^, it is possible that HEMK1 and HEMK2 have different preferences for substrate binding.

Given the potential difference in substrate recognition, we thought that HEMK1 might modify GGQ-containing proteins other than eRF1 (targeted by HEMK2). The apparent candidates were four potential mtRFs—MTRF1, MTRF1L, MTPL58, and MTRFR^20–22^. All four mtRFs contained the conserved GGQ motif, while only MTRF1 and MTRF1L possessed a peptide chain release factor domain (Pfam: PCRF, PF03462) (Fig. 1C,D). Permutation array of enzyme–substrate binding, using the sequence of eRF1 (position 179–192), revealed that G-Q-X3-R was the minimal recognition element for HEMK2, with the + 4 position of the GGQ motif (R189) being absolutely essential^16^ (Figure S2B). However, the arginine residue was not found at the + 4 position of the GGQ motif in any of the four human mtRFs (Figures S2B,S3), suggesting it is unlikely that HEMK2 is the methyltransferase responsible for human mtRFs.

### HEMK1 is responsible for glutamine methylation of mtRFs

**Figure 2:**
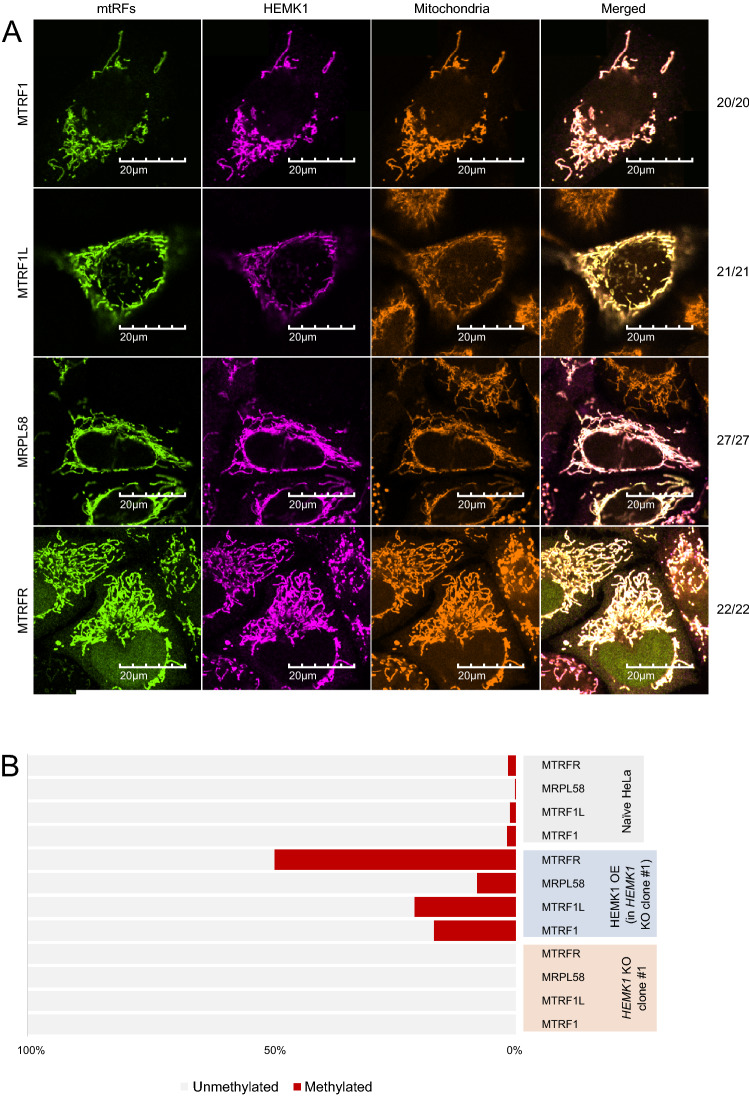

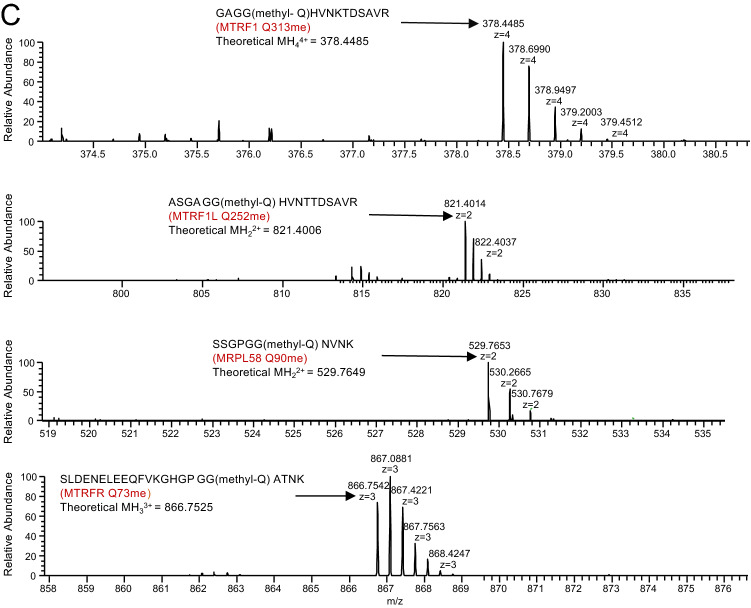
Glutamine methylation in the “GGQ” motif of four mtRFs mediated by HEMK1. (**A**) Subcellular localization of transiently expressed exogenous HEMK1 and mtRFs in HeLa cells. Representative images of the cell expressing both HEMK1-cFLAG and individual mtRF-cHA were shown. The number of cells showing the colocalization of mtRF, HEMK1, and MitoTracker per the number of analyzed cells are shown in the right side of the panels. Cells with both HEMK1-cFLAG and individual mtRF-cHA co-expressed were analyzed. Note that, although some cells showed mtRFs-cHA and/or HEMK1-cFLAG in the nucleus possibly because of too-much expression of transgene(s), we excluded these cells for quantification to avoid collecting abnormal phenotypes. HEMK1-cFLAG, magenta; mtRFs-cHA, green; MitoTracker Red, orange. Scale 20 μm in total length. (**B**) Approximate methylation efficiency. Stably expressed exogenous mtRFs-cHA in naïve HeLa, *HEMK1* KO clone #1, and *HEMK1* KO clone #1 complemented with stably expressed HEMK1-cFlag. For fragment counts and methylation ratio of each methylated peptide, see Figure S5A. (**C**) MS1 spectra of mtRFs. Theoretical mass corresponds to specific methylated mtRFs peptides. See Table S2 for detailed MS2 spectra and chromatograms of methylation of each sites.

Although serval reports have demonstrated the mitochondrial localization of HEMK1 and mtRFs^13,20–22,25^, co-localization of HEMK1 and mtRFs has yet been shown. In order to determine whether HEMK1 methylates the mtRFs, we first confirmed the subcellular localization of both exogenously expressed enzymes and substrates. We co-expressed HA-tagged mtRFs individually with FLAG-tagged HEMK1 and performed immunofluorescence imaging to track their localization. As expected and consistent with previous reports, HEMK1 and all four mtRFs were localized in the mitochondria (Fig. 2A).

The purified recombinant PrmC could induce the methylation of bacterial RF1 in vitro^7^. Therefore, we attempted to detect the catalytic activity of recombinant HEMK1 against mtRFs in vitro; however, we did not observe any methyltransferase activity (data not shown). Hence, we decided to monitor the methyltransferase activity of HEMK1 in cells. We generated *HEMK1* KO HeLa cells via CRISPR-Cas9-mediated gene deletion. This deletion removed both the SAM-binding and substrate-binding motifs of HEMK1, resulting in a non-functional enzyme (Figure S4). To monitor the impact of HEMK1 depletion on the methylation of mtRFs, we expressed HA-tagged mtRFs individually in the naïve or *HEMK1*-KO HeLa cells and immune-purified the proteins for mass spectrometry analysis to detect methylated peptides. In naïve HeLa cells, we found glutamine methylation at Q252 of MTRF1L, Q313 of MTRF1, Q90 of MRPL58, and Q73 of MTRFR, within their GGQ motifs (Fig. 2B,C; Table S2). In contrast, these glutamine methylation events were not detected in the *HEMK1* KO cells (Fig. 2B, Figure S5A). Exogenous expression of HEMK1-cFLAG in the *HEMK1* KO cells rescued and in fact, enhanced the methylation of all four mtRFs, compared to that in the naïve cells (Fig. 2B, Figure S5A).

To further clarify whether glutamine methylation of mtRFs is directly caused by the enzymatic activity of HEMK1, we generated a mutant HEMK1 with a Y242E substitution at the NPPY substrate-binding motif, which was predicted to inactivate the enzymatic activity. HEMK1^Y242^^E^ displayed the same mitochondrial localization as the wild-type HEMK1 (Figure S5C). However, the glutamine methylation of MTRF1L in *HEMK1* KO HeLa cells was not induced by the HEMK1^Y242E^ mutant (Figure S5A). These data further support that HEMK1 is a glutamine methyltransferase for all the four mtRFs.

### Loss of *HEMK1* has minimal impact on HeLa cell physiology

**Figure 3:**
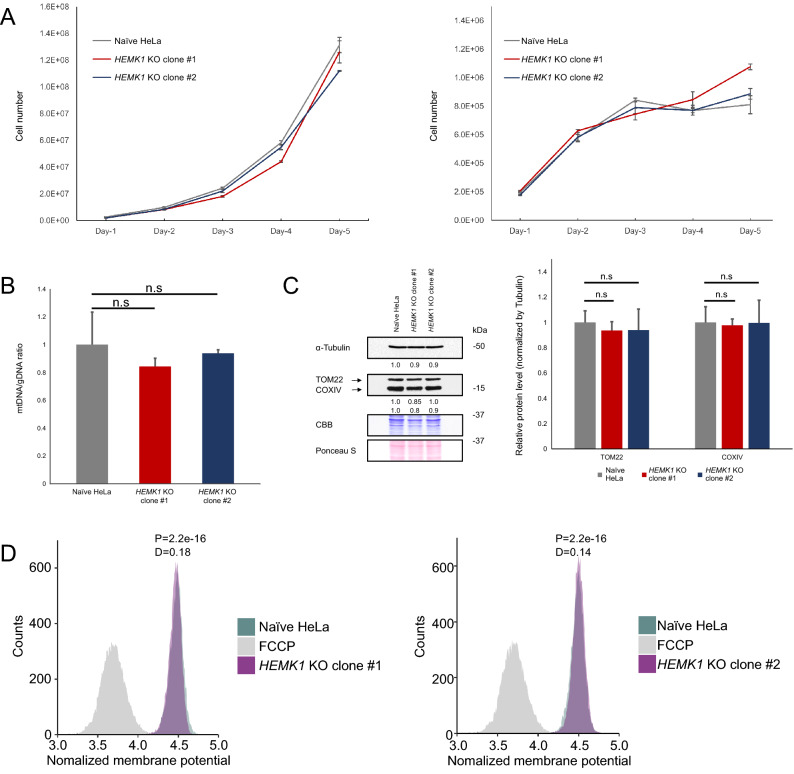
Impacts of *HEMK1* depletion on cell growth and mitochondrial properties. (**A**) Cell growth in high-glucose and galactose media. Growth curve of naïve HeLa and *HEMK1* KO clones #1 and #2 in different media. Left panel: high-glucose media; right panel: galactose media. The numbers represent a mean ± SEM from three independent samples. (**B**) Assessment of relative mtDNA amounts (mtDNA/gDNA) by qPCR. Data were normalized to gDNA abundance. Data represent a mean ± SEM from three independent samples. The Student’s t-test, two-tail, was used for testing the statistical significance. *n.s* not significant. (**C**) Western blot of mitochondrial outer membrane protein TOM22, and inner membrane protein COXIV from naïve HeLa and *HEMK1* KO clones (#1 and #2). Left panel shows representative western blot result. α-Tubulin was used as a loading control. CBB and Ponceau S staining were shown as loading and transfer indicators. Numbers shown at the bottom of each panel indicated the normalized intensity referring to signals from naïve HeLa cells. Right panel: quantitation of mitochondrial membrane protein TOM22 and COXIV Western blot signals with a mean ± SEM from four independent samples. The Student’s t-test, two-tail, was used for testing the statistical significance. *n.s* not significant. Biological repeats were shown in Figures S6C and S9A–F. (**D**) Assessment of mitochondrial properties by FACS analysis. Mitotracker Red CMXRos signal was normalized with Mitotracker Green FM signal (see “Materials and methods” section for the details). Data from 10,000 cells are depicted. The Mann–Whitney *U*-test, two-tail, was used for testing the statistical significance (P value). The Cohen’s d-test was used for testing the effect size (D value).

Since the loss of PrmC in K-12 *E. coli* causes growth retardation^6^, we assessed the impact of the loss of *HEMK1* on cell growth. Under the fermentative culture condition with glucose as a carbon source, we monitored the growth of naïve and *HEMK1* KO HeLa cells, but did not find a clear difference between them (Fig. 3A, left). We also assayed under the respiratory condition with galactose as a carbon source, but HEMK1 depletion did not show clear impact on the cell growth (Fig. 3A, right).

It has been shown that mitochondria DNA (mtDNA) replication is a highly dynamic process that is linked to mitochondrial properties including total mass and activities^26^. We thus examined the potential impact of HEMK1 depletion on mtDNA copy number. To this end, we performed a quantitative polymerase chain reaction using total DNA extracts to assess mtDNA amounts (as a ratio to genomic DNA [gDNA]). Again, we failed to detect an apparent difference between naïve and *HEMK1* KO cells regarding the mtDNA-gDNA ratios, suggesting that the mtDNA replication is likely not affected by HEMK1-mediated glutamine methylation on mtRFs (Fig. 3B).

We then examined the impact of HEMK1 depletion on mitochondrial membrane protein contents. Potential changes in one of the mitochondrial outer membrane proteins, TOM22, and one of the mitochondrial inner membrane proteins, COXIV, were investigated. However, the western blot analysis of these mitochondrial proteins revealed no changes in protein expression in *HEMK1* KO cells (Fig. 3C, Figure S6C).

It has been shown that mitochondria DNA (mtDNA) replication is a highly dynamic process that is linked to mitochondrial properties including total mass or activities^26^. Therefore, we measured mitochondrial membrane potential via a flow cytometry-based assay. Here, we used MitoTracker Red, a membrane potential sensitive dye^27^. To control the effect of mitochondrial volume, we used membrane potential insensitive MitoTracker Green, which stains the mitochondrial membrane. Although the loss of *HEMK1* significantly affected (P value = 2.2e−16) the signals of MitoTracker Red (Fig. 3D), the change was nearly negligible (D value < 0.2) in both clones. Thus, the impact on the net mitochondrial membrane potential (normalized MitoTracker Red signal) was marginal (Fig. 3D).

### HEMK1-mediated mtRF methylation could be dispensable in mitochondrial translation under standard culture conditions

**Figure 4:**
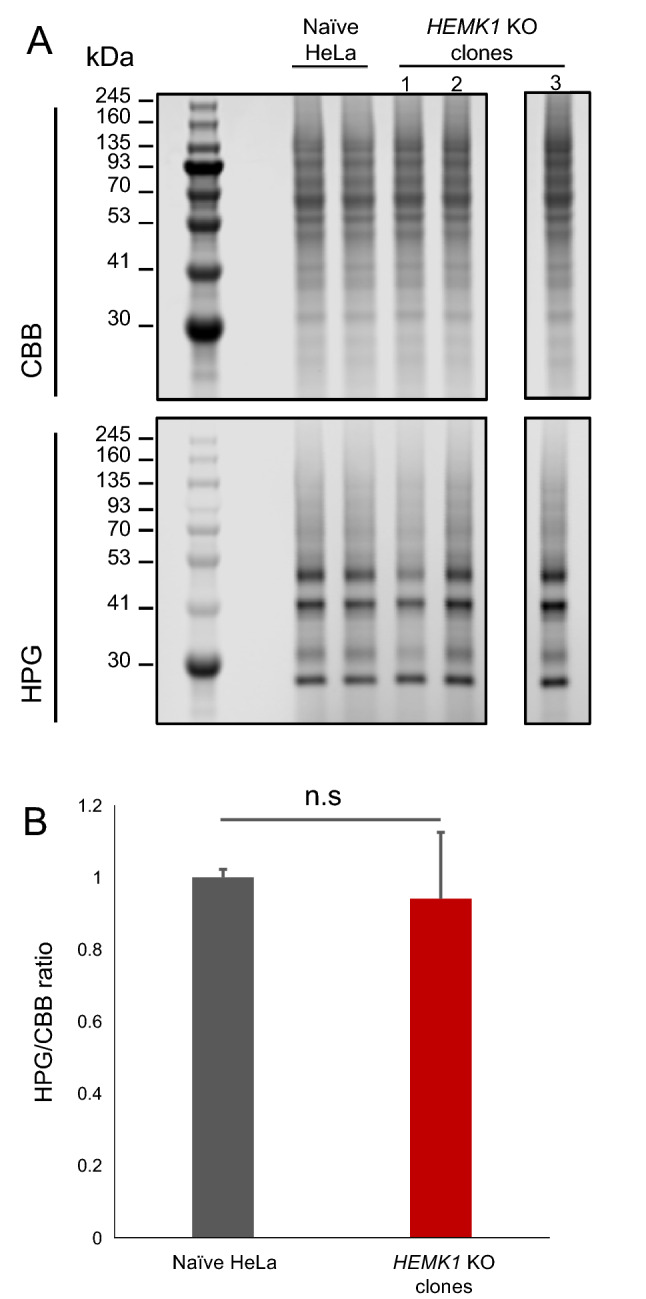
The assessment of mitochondrial translation in *HEMK1* KO cells. (**A**) Representative gel images of total and nascent mitochondrial proteins. Infrared (IR)-800 dye was conjugated to HPG-containing newly synthesized proteins. Total protein was stained with CBB. A black line is inserted between lanes 4 and 5 (counting from the left-hand side) because they were not adjacent lanes, although these lanes are originated from the same gel. The original image is shown in Figure S6A. (**B**) Bar graph of HPG signal/CBB signal for (**A**). Error bars represented the mean ± SEM calculated from samples shown in Figure S6A (see detailed measurements in Figure S6A). The Student’s t test, two-tail, was used for testing the statistical significance.

Even with the limited physiological influence of HEMK1 depletion in mitochondria, we extended our analysis to mitochondrial translation. To directly examine the impact of HEMK1 depletion and the role of HEMK1-mediated mtRFs methylation in mitochondrial translation, we performed fluorescent noncanonical amino acid tagging for mitochondrial translation (mito-FUNCAT)^28,29^ to assess nascent protein synthesis in *HEMK1* KO cells. Out of four *HEMK1* KO clones, only KO clone #1 showed a decrease in nascent protein synthesis (Fig. 4A,B, Figure S6A,B), suggesting that this reduction stemmed from clonal variation and not HEMK1 depletion. To further ascertain this, we performed a rescue experiment using the wild-type HEMK1 and the HEMK1^Y242^^E^ mutant. As expected, the exogenous expression of neither wild-type HEMK1 nor HEMK1^Y242E^ mutant could reverse the changes in nascent protein synthesis in *HEMK1* KO clone #1 (Figure S7A,B). Furthermore, we performed western blot of an mtDNA-encoding protein MT-CO1. Similar to that in the mito-FUNCAT assay, *HEMK1* KO clone #1 showed lower MT-CO1 levels, and this phenotype was unrelated to *HEMK1* expression (Figure S7C,D). Therefore, we concluded that *HEMK1* inactivation does not have a clear impact on mitochondrial translation.

## Discussion

Ishizawa et al*.* reported HEMK1-mediated methylation (14 Da addition) of the MTRF1L-derived peptide^13^; here, we extended their findings onto other mtRFs and demonstrated that HEMK1 could methylate glutamine in the GGQ motif of all the four mtRFs. However, our analysis, which utilized exogenously expressed mtRFs, posed an analytic hurdle in the assessment of the methylation level of endogenous mtRFs. Since we could not access to good antibody to detect HEMK1 protein, we compared RNA expression level of *HEMK1* in naïve Hela (endogenous one) vs. the *HEMK1* KO clone #1 complemented with exogenous *HEMK1* (exogenous HEMK1 cDNA only), which was used for exogenous mtRFs GGQ motif methylation analysis shown in Fig. 2B,C and Fig. S5A. Exogenous *HEMK1* was highly expressed (> 700 times higher than endogenous one in naïve Hela, Fig S5B) in this complemented *HEMK1* KO cells. However, with such high *HEMK1* expression, mtRFs methylation was hardly reaching 50%. Given that the exogenously expressed mtRFs in naïve HeLa cells were methylated at a low frequency (Fig. 2B, Figure S5A), the expression levels of *HEMK1* in HeLa cells might be insufficient for full methylation of endogenous mtRFs. Another possibility for the incomplete methylation of mtRFs is that although HEMK1 is the enzyme responsible for glutamine methylation, other cofactor(s) may be needed for the catalytic reaction, as seen in HEMK2 homologs^14^, which might also explain why recombinant HEMK1 did not show enzymatic activity in the in vitro assay. Therefore, even if a sufficient amount of HEMK1 was exogenously supplied (Figure S5B), the amount of such putative endogenous cofactor(s) might restrict this enzymatic reaction in cells. In any case, if any approach for investigating GGQ methylation of endogenous mtRFs is accessible, such as, specific antibodies capable of immunoprecipitation of mtRFs or the epitope tagging to the endogenous mtRFs, it will be possible to enrich the endogenous ones and clarify their GGQ methylation status. Furthermore, as described, we have not succeeded in detecting the methylation of Q by HEMK1 in vitro. If the HEMK1-mediated Q methylation reaction can be established in vitro, the details of the enzymatic reaction of HEMK1 and its regulatory mechanism could be further elucidated.

Given that the loss of *PrmC* in *E. coli* K-12 strain leads to growth retardation^6,7,30^, it is possible that the loss of methylation of mtRFs could cause similar biological consequences. Unfortunately, we could not identify any clear mitochondria-associated phenotypes in terms of cell growth, mitochondrial DNA replication, membrane potential, or even mitochondrial translation, a biological reaction directly supported by mtRFs. Methylated bacterial RFs can just accelerate the termination reaction, and this methylation is not essential at the termination step, as hydrolysis still occurs, although at a slower rate^9^. Again, the lower rate of mtRF methylation seen in HeLa cells suggests the limited role of mtRFs in mitochondrial protein synthesis. The restricted function of GGQ methylation in mtRFs is also supported by the Genome Aggregation Database^31^, which shows the probability of "being the loss-of-function intolerant" score of *HEMK1* as 0, suggesting that *HEMK1* is unlikely an essential gene*.*

Although our results showed that HEMK1 depletion plays a limited role in translation, further analysis is required for clarifying any biological role of HEMK1-mediated GGQ methylation, especially a comprehensive molecular understanding of the four putative mtRFs, which has been long contested. mtRF1L is a canonical release factor with peptidyl-tRNA hydrolysis activity in ribosomes^22^. Furthermore, such activity has also been reported in MRPL58 (ICT1)^32^, but the role in termination is still a matter of debate^33,34^. A recent structural study showed that MRPL58 recognizes the vacant A site in ribosomes translating truncated mRNA^23^, suggesting that MRPL58 may rescue the stalled ribosomes on the ends of aberrant mRNAs, rather than participate in the standard termination reaction on stop codons. Moreover, Ramakrishnan and colleagues reported that MTRFR, associated with a split large subunit of the mitoribosome, serves as a quality control factor and cleaves the peptidyl-tRNAs halted in the middle of elongation^24^. In addition to termination and ribosome recycling, MRPL58 may also plays an important role in mitoribosome biogenesis or basal stability, as this protein is an integral constituent of the large subunit of the mitoribosome^20,35,36^. Further functional analysis of mtRFs in mitochondrial translation will help in enhancing the understanding of HEMK1-mediated methylation.

## Materials and methods

### Cell culture

HeLa cells were obtained from RIKEN BioResource Research Center (BRC) and cultured in standard Dulbecco's modified Eagle' medium (DMEM high-glucose 4.5 g/l, Nacalai) with 10% FBS (Biosera), 1 × glutamine (Gibco), and 1 × Penicillin–Streptomycin (Gibco) at 37 °C humidified incubators with 5% CO_2_ supplied. For cell growth experiment under the respiratory condition, we used modified Dulbecco's modified Eagle' medium (No Glucose, Nacalai) with the addition of 4.5 g/l D(+)-galactose (Wako), 10% dialyzed FBS (Thermo Fisher Scientific), and 1 × Penicillin–Streptomycin (Gibco). The medium was changed routinely every 48 h. For the cell counting experiment, 0.5 million cells were seeded on a 10-cm dish for cell counting experiment. Cell counts started the next day after seeding for five consecutive days.

### Sequence alignment, structure analysis, and phylogenetic analysis

Amino acid sequences were retrieved from UniProt (https://www.uniprot.org/). Alignments were generated using ClustalOmega (https://www.ebi.ac.uk/Tools/msa/clustalo/) with default settings. Alignments editing, color-coding, and phylogenetic analysis (by BLOUSUM62 matrix) were performed using the Jalview program. Domain organizations were retrieved from Pfam^37^ database (http://pfam.xfam.org/). HEMK1/2 sequence-specific substrate binding sites were generated by WebLogo (http://weblogo.threeplusone.com/)^38^. DeepMito, deep-learning approaches trained with dataset comprising known mitochondrial proteins, (http://busca.biocomp.unibo.it/deepmito/)^39^ and MitoFates, amino acid composition and physiochemical properties-based analysis, (http://mitf.cbrc.jp/MitoFates/cgi-bin/top.cgi)^40^ were used for predictions of mitochondrial targeting sequences. Disordered region and secondary structure prediction were conducted using PSIPRED (http://bioinf.cs.ucl.ac.uk/psipred/)^41^. Sequence alignments with secondary structure elements were generated by ESPript (http://espript.ibcp.fr/ESPript/ESPript/)^42^. All UniProt protein IDs used in this study are listed in Table S1.

### DNA construction and establishment of *HEMK1* KO cells

*HEMK1* KO cell lines were generated by CRISPR-Cas9 mediated gene knockout. In brief, two sgRNAs (targeting *HEMK1* exon 5 and exon 8) were cloned into pL-CRISPR.EFS.tRFP (Addgene #57819)^43^ and pKLV2-U6gRNA5(BbsI)-PGKpuro2ABFP-W (Addgene #67974)^44^, respectively. After verification by Sanger sequencings, naïve HeLa cells were transfected with both sgRNA containing plasmids by Lipofectamine 2000 (Thermo Fisher Scientific) as instructed by the manufacturer. At 48 h post-transfection, HeLa cells were sorted by flow cytometry for RFP and BFP dual positive populations. Generally, 200–300 cells were seeded onto a 6-cm dish and cultured until visible colonies were seen. Single clones were then expanded in a 24-well dish. *HEMK1* KO clones were confirmed by PCR. For *HEMK1* KO clone #3 and #4, Sanger sequencing was also performed because large genomic deletion was only found on one allele. See Table S1 for the sequencing results.

DNA fragments encoding four mtRFs were PCR-amplified from HeLa total cDNA using specific primers. DNA fragments encoding mtRFs were cloned into EcoRI-linearized pQCXIP vector (Clontech) by In-Fusion cloning (Clontech) with HA tag at the C-terminal of mtRFs. DNA fragment encoding *HEMK1* cDNA was PCR-amplified from HeLa total cDNA by specific primers, and the PCR fragments was cloned into pcDNA3-cFLAG (Thermo Fisher Scientific) using EcoRI and NotI double digestion. *HEMK1* mutant was generated by site-directed mutagenesis with Phusion polymerase (Thermo Fisher Scientific). For transient expression (HEMK1, HEMK1^Y242^^E^, and mtRF1L), HeLa cells were transfected using Lipofectamine 2000 (Thermo Fisher Scientific) followed the protocol from the manufacturer. At 72 h post-transfection, cells were harvested with ice-cold PBS, and then lysed in RIPA buffer for SDS-PAGE analysis.

### Stable cell line establishment

*HEMK1* wild-type and the mutants were subcloned from pcDNA3-cFLAG (Thermo Fisher Scientific) constructs into EcoRI-linearized pQCXIN (Clontech) by In-Fusion cloning (Clontech). Viral particles were produced in HEK293T cells (cultured at the same conditions as HeLa cells) by pQCXIN/P and the Retroviral Expression System (Clontech). The medium was replaced on the next day after transfection. Viral particles were collected at 48 h post-transfection and filtered by 0.45-μm membrane (EMD). HeLa cells were infected by the viral particles (with 8 μg/ml polybrene, Sigma). At 24 h post-infection, cells were selected with neomycin (500 μg/ml for up to 14 days). Stable cell lines expressing mtRFs were generated similarly using the former established *HEMK1* wild-type/mutants cell lines (selected with 2 μg/ml puromycin for 3 days). Primers and vectors used in this study were listed in Table S1.

### Western blot analysis

Cells harvested using ice-cold PBS (with 1 × protease inhibitor cocktail [Nacalai] and 1 mM PMSF), were lysed by RIPA buffer (with 1 × protease inhibitor cocktail [Nacalai] and 1 mM PMSF) on ice for 5 min. Sonication was performed on ice subsequently. The lysate was centrifuged at 10,000*g,* 4 °C for 10 min. The supernatant was transferred to a new tube, and protein concentration was measured using the Bradford Protein Assay Kit (BioRad Laboratories). The lysate was denatured by NuPAGE LDS Sample Buffer (Thermo Fisher Scientific) with 1 mM 2-mercaptoethanol at room temperature. Proteins were separated by SDS-PAGE and transferred onto PVDF membrane (Immobilon-FL-PVDF, Millipore). For imaging, both X-ray film (FUJIFILM) with chemiluminescence and ODYSSEY CLx (LI-COR Biosciences) with infrared fluorescence were used. In brief, PVDF membrane was blocked using TBS-T (1 × TBS and 0.1% Tween-20) with skimmed milk at room temperature for 1 h, followed by incubation with the primary antibody in TBS-T with 3% BSA at 4 °C overnight with mild shaking. The membrane was washed with TBS-T for 10 min twice before secondary antibody incubation. In the case when infrared fluorescence was used for detection, Tween-20 was omitted from the blocking buffer, and secondary antibody was used in the dark for 1 h at room temperature. Antibodies used in this study are listed in Table S1.

### Immunofluorescence microscopy analysis

For the mtRFs and HEMK1 co-localization analysis in mitochondria, 5 × 10^5^ of HeLa cells were reverse transfected using Fugene HD (Promega) with 0.5 μg each of HEMK1-cFLAG WT and individual mtRFs-cHA expressing plasmids followed the protocol from the manufacturer. For the HEMK1^Y242E^ Mut cellular localization experiment, 0.5 μg HEMK1-cFLAG WT or HEMK1^Y242E^ Mut expressing plasmid was used for transfection. The transfected cells were grown on sterilized cover glasses for 24 h. Then, the cells were first washed with PBS then fixed using 4% PFA at 37 °C for 10 min. Cells were washed with PBS twice, followed by permeabilization using PBS with 0.2% Triton X-100 for 10 min at room temperature. Then, cells were blocked with 3% goat serum in PBS at room temperature for 30 min. Cells then incubated with primary antibodies at 4 °C, overnight. Secondary antibodies were administered as guided by manufacturers. MitoTracker Red CMXRos reagent (200 nM, Thermo Fisher Scientific) was added directly into the media at 30 min before PFA fixation for mitochondria staining. Slides were mounted on slide glasses (Matsunami) with Prolong with DAPI mounting reagent (Thermo Fisher Scientific). Images were captured with 60× objective lens and processed by Olympus FV3000 system and its suite software. Exogenous HEMK1-cFLAG and mtRFs-cHA Double-expressing cells were selected for quantitation. Quantitation was described as x/y, where x was the number of cells showing the colocalized phenotype, y was the total number of the examined cells in which (1) both HEMK1-cFLAG and individual mtRF-cHA were co-expressed and (2) none of mtRFs-cHA and HEMK1-cFLAG were not detected in the nucleus in order to exclude abnormal phenotypes by too much expression of transgene(s). Antibodies used in this study are listed in Table S1.

### Detection of mitochondrial membrane potential by flow cytometry

Experimental procedures, signal visualization, and normalization were adapted from previous studies with minor modifications on the cell seeding step^45–47^. Cells (naïve HeLa, *HEMK1* KO clone #1, and *HEMK1* KO clone #2) were seeded on 6-cm dishes at ~ 70% confluency. Then, after 16 h cell culture (not confluent), the cells were first washed with PBS once and then replaced with Mitotracker Red CMXRos (200 nM, Thermo Fisher Scientific) and Mitotracker Green FM (150 nM, Thermo Fisher Scientific) containing PBS with 5% FCS. Cells were incubated at 37 °C for 25 min. Then, cells were dissociated from the dishes by 0.5% trypsin and neutralized by PBS with 5% FCS. Cells were pelleted and resuspended in 0.3 ml of PBS with 5% FCS for flow cytometry analysis (BD FACSAria II, BD Biosciences). The value of Mitotracker Green FM was standardized by the mean of naïve HeLa, *HEMK1* KO clone #1, and *HEMK1* KO clone #2 experiments (10,000 cells data each). Then the value of Mitotracker Red CMXRos in each cell was normalized by the standardized value of Mitotracker Green FM from the cell. The statistical significance (P value) was calculated by the Mann–Whitney *U*-test, two-tail. The effect size (D value) was calculated by the Cohen's D test.

### Immunoprecipitation for mass spectrometry (MS) analysis

Stable mtRFs and mtRFs with HEMK1 (co-)overexpressing cells were first lysed on ice for 10 min using lysis buffer A (50 mM Hepes pH 7.5, 140 mM KCl, 10% glycerol, 0.5% NP40, 1 × protease inhibitor cocktail [Nacalai], and 1 mM PMSF). The lysate was then sonicated with Ultrasonic Disruptor (TOMY UR-21P) with 70% output for 10 pluses on ice and precleared using Protein G Sepharose 4 Fast Flow beads (GE Healthcare) at 4 °C for 30 min with mild rotation. The supernatant was collected by 10,000 g spinning at 4 °C for 10 min and subsequently incubated with anti-HA antibody at 4 °C for 1 h and then with Protein G beads for 2 h. Then, beads were collected at 500*g* centrifugation and washed once with lysis buffer A and three times with bead wash buffer (20 mM Tris 6.8, 150 mM NaCl, 10% glycerol, and 1 mM PMSF). The beads were boiled in 1 × Laemmli buffer at 95 °C for 5 min for elution. Eluted proteins were separated on 15% Tris–glycine gel with MOPS buffer. Gels were stained using the Pierce Silver Stain for Mass Spectrometry kit (Thermo Fisher Scientific), and regions corresponding to different mtRFs were excised for mass spectrometry.

### MS analysis

The excised gels were destained as instructed by the manufacturer. The proteins were digested in gel with trypsin (TPCK-treated, Worthington Biochemical Corporation). The peptide mixture was separated on a nanoflow LC (Easy nLC 1200; Thermo Fisher Scientific, Waltham, MA) using a nano-spray column (NTCC analytical column, C18, φ75 μm × 150 mm, 3 μm, Nikkyo Technos) with a linear gradient of 0–100% buffer B (80% aqueous acetonitrile containing 0.1% formic acid) at a flow rate of 300 nl/min over 20 min. The elution was sprayed online to a Q Exactive HF-X mass spectrometer (Thermo Fisher Scientific) equipped with a nano-spray ion source. The MS/MS data were acquired in a Top10 data-dependent manner. Proteins were identified using Proteome Discoverer 2.2 (Thermo Fisher Scientific) with MASCOT program 2.6 (Matrix science) using an in-house database. The mass spectra and mass chromatograms were drawn by Qual Browser 4.1.50 (Thermo Fisher Scientific).

### qPCR analysis

MT-ND2 and ALU repeats were selected for quantification of mtDNA and gDNA content, respectively. Total DNA was extracted with the Nucleospin DNA isolation kit (Macherey Nagel). One nanogram of total DNA was used for qPCR with Power SYBR Green PCR master mix (Thermo Fisher Scientific). Step one plus real-time PCR (Applied Biosystems) was performed according to the manufacturer's instruction. Data were processed and plotted with Microsoft Excel. For primers used in qPCR, see Table S1.

### On-gel mito-FUNCAT assay

Cells (naïve HeLa, *HEMK1* KO clone #1, clone #2, and *HEMK1* KO clone #1 rescued lines) were grown on 6-well dish in standard culture media (-antibiotics) until the day of assay. Cells were washed with protein labelling media (DMEM [ThermoFisher Scientific], 10% FBS, 48 mg/ml l-cystine dihydrochloride [Nacalai], and 862 mg/ml l-alanyl-l-glutamine [Nacalai]) once and then incubated in methionine-free medium with L-homopropargylglycine 50 μM (Jena Bioscience) and anisomycin 100 μg/ml (Alomone Labs) for 4 h at 37 °C humidified incubators with 5% CO_2_ supplied. Cells were lysed with ice-chilled lysis buffer (20 mM Tris–HCl pH 7.5, 150 mM NaCl, 5 mM MgCl_2_, 1% Triton X-100). Lysates were cleared by centrifugation for 10 min at 20,000*g*, 4 °C. The supernatants were used for CLICK reaction with Click-iT Cell Reaction Buffer Kit (ThermoFisher Scientific) and IRdye800CW Azide (LI-COR Biosciences), according to the manufacturer's instruction. After the CLICK reaction, free IRdye800CW Azide was removed using illustra MicroSpin G-25 Column (GE Healthcare) equilibrated with lysis buffer containing 1 mM DTT. Samples were denatured at 50 °C with 4 × Protein Loading Buffer (LI-COR Biosciences) with 10% 2-mercaptoethanol (Nacalai). Samples were loaded onto the polyacrylamide gel (NuPAGE, 4 to 12%, Bis–Tris [ThermoFisher Scientific]) with 5 μl protein ladder marker (NIPPON Genetics). Electrophoresis was conducted in 1 × MES buffer (ThermoFisher Scientific) for 22 min at 200 V constant. The gel was fixed with fixation reagent (40% ethanol, 10% acetic acid, and 50% ddH_2_O) for 15 min at room temperature. After fixation, the gel was destained with MilliQ water and subsequently imaged by Odyssey CLx (LI-COR Biosciences) with IR800 channel for the newly synthesized mitochondrial polypeptides. For the total protein imaging, the same gel (after IR800-channel imaging) was incubated with GelCode Blue reagent (ThermoFisher) for 15–60 min then destained with MilliQ water. The destained gel was imaged by Odyssey CLx (LI-COR Biosciences) with IR700 channel.

## Supplementary Information

Supplementary Information 1.

Supplementary Information 2.

Supplementary Information 3.
