# Supplementary material for: Mammalian HEMK1 methylates glutamine residue of the GGQ motif of mitochondrial release factors
Source: Sci Rep. 2022 Mar 8;12:4104. doi: 10.1038/s41598-022-08061-y (PMC8904536; doi:10.1038/s41598-022-08061-y)

Mammalian HEMK1 methylates glutamine residue of the GGQ motif of mitochondrial release factors

Qi Fang, Yusuke Kimura, Tadahiro Shimazu, Takehiro Suzuki, Ayumi Yamada, Naoshi Dohmae, Shintaro Iwasaki, and Yoichi Shinkai

Supplementary Figure and Table legend

Figure S1. Sequence alignment of various PrmC family proteins.

Complete sequence alignment of Figure 1B.

Figure S2. DISOPRED and PSIPRED prediction of bacterial PrmC, HEMK1, and HEMK2 and sequence composition of GGQ flanking region of various RFs.

A) The left panels are the disordered structure prediction of bacterial PrmC, HEMK1, and HEMK2 by DISOPRED3 in PSIPRED. The cutoff for disorder was 0.5 (dashed line). The right panels are the predicted secondary structures and properties calculated by PSIPRED 4.0.

B) Consensus logo of flanking region of bacterial RF1, human mtRFs, and various eRF1 across different species. A GQX3R (the arginine residue is boxed in red) motif is required for the HEMK2 binding [16].

Figure S3. Sequence alignment of bacterial RF1 and human mtRFs.

Complete sequence alignment of Figure 1D.

Figure S4. Knockout scheme of *HEMK1* with CRISPR-Cas9 system.

A) Exon 5–8 of *HEMK1* was removed by two sgRNAs. Primer 1, 2, 3, and 4 were used for PCR screening.

B) *HEMK1* KO clones were confirmed by genomic DNA PCR. Primer set A (left panel) : Primer 1+3+4. Primer 1+3 = 1358bp (WT band). Primer 1+4 = 832bp (KO band). Primer set B (right panel) : Primer 2+3 = 523bp (WT band). Original images of Primer set A and B were shown in Figure S10A and Figure S10B.

C) Depiction of genomic DNA excision (or small deletion) of *HEMK1* KO clones. Sanger sequencing results of *HEMK1* KO clones, see Table S1.

Figure S5. Fragment counts of MS analysis, *HEMK1* expression level in naïve HeLa and *HEMK1* KO clone #1 Res. cells, and subcellular localization of HEMK1^{Y242E} mutant.

A) Fragment counts of MS analysis related to Figure 2B.

B) RT-qPCR of HEMK1 expression level in Naïve HeLa and *HEMK1* KO clone #1 Res. cells. Error bars represented the mean ± SEM calculated from 4 technical repeats.

C) Comparison of subcellular localization of HEMK1-cFLAG wild-type (WT) and HEMK1^{Y242E}-cFLAG mutant. The number of cells showing the colocalization of HEMK1 and MitoTracker per the number of analyzed cells are shown in the right side of the panels. Cells with HEMK1-cFLAG expressed were analyzed. Note that, although some cells showed HEMK1-cFLAG in the nucleus possibly because of too-much expression of transgene, we excluded these cells for quantification to avoid collecting abnormal phenotypes. HEMK1-cFLAG, magenta; MitoTracker Red, orange. DAPI, gray. Scale: 20 µm in total length.

Figure S6. Quantification of newly synthesized mitochondrial proteins and biological repeats of mitochondrial membrane protein western blots.

A) Quantification of on-gel mito-FUNCAT for Figure 4A. Four naïve HeLa lanes and six *HEMK1* KO clone lanes were used for the bar graph in Figure 4B.

B) Quantification of on-gel mito-FUNCAT for Figure S7A. WT-Res, wild-type HEMK1 expression; Mut-Res, HEMK1^{Y242E} mutant expression. The experiment was done in triplicate. Data from this figure was used for the bar graph (N=3) in Figure S7A.

C) Biological repeats of Figure 3C; signals were measured by ImageJ and plotted in Figure 3C (right panel).

Figure S7. Rescue experiment for *HEMK1* KO clone #1 and assessment of MT-CO1 protein abundance by western blot.

A) Same as Figure 4A.

B) Same as figure 4B. The Student's t-test, two-tail, was used for testing the statistical significance.

C) The western blot images (detected by LI-COR) for MT-CO1 (encoded in mitochondrial DNA). The signals of COXIV, encoded in nuclear DNA, were used for normalization.

D) Bar graph of quantification of A (MT-CO1/COXIV). Data represent a mean ± SEM from 4 independent repeats. The Student's t-test, two-tail, was used for testing the statistical significance.

Figure S8. Original images of Figure 3C (left panel).

A) and B) were the same membrane but with different exposure.

C) was the CBB stained gel with the same sample loading amount of A), B) and D).

D) was the Ponceau S. staining of membrane for A) and B).

Figure S9. Original images of the quantitation bar graph in Figure 3C (right panel) and Figure S6C.

A), C), and E) were the original image for Figure S6C (left panel).

B), D), and F) were the original image for Figure S6C (right panel).

Figure S10. Original images of the Figure 4A and Figure S7A.

Same with Figure S6A and S6B, but without additional description.

A) was the original image of Figure S6A.

B) was the original image of Figure S6B.

Figure S11. Original images of the DNA electrophoresis gel of Figure S4B.

A) was the original image of Figure S4B, Primer set A.

B) was the original image of Figure S4B, Primer set B.

Table S1. Primers, vectors, antibodies, Protein ID, mitochondrial localization signal predictions and sequencing results of *HEMK1* KO clones.

Table S2. MS/MS spectra, chromatograms, and reference sequences of methylated mtRFs peptide fragments.

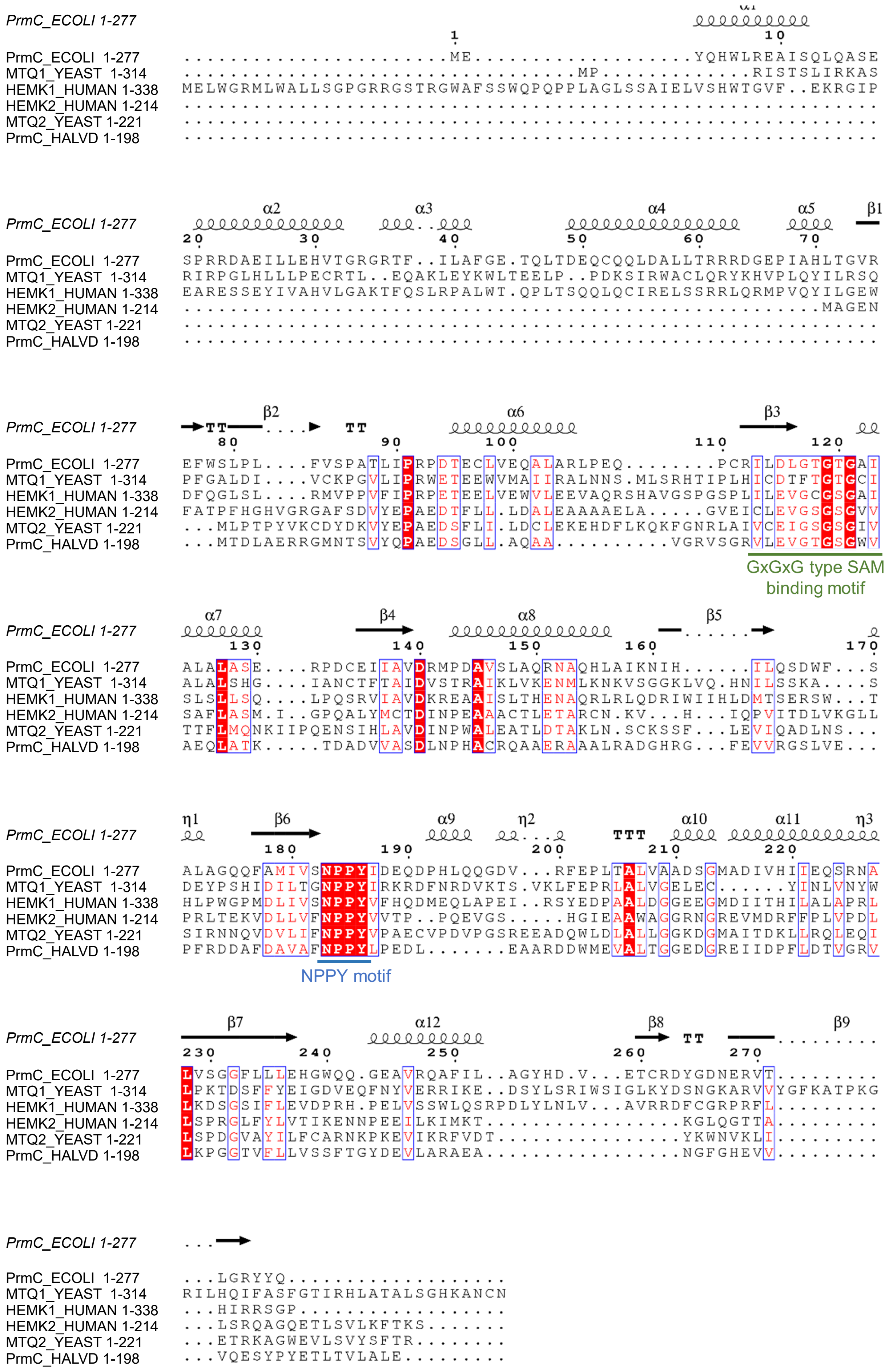
PrmC_ECOLI 1-277
α1
1 10
PrmC_ECOLI 1-277 ....ME...................YQHWLREAISQLQASE
MTQ1_YEAST 1-314 ....MP..............RISTSLIRKAS
HEMK1_HUMAN 1-338 MELWGRMLWALLSGPGRRGSTRGWAFSSWQPQPPLAGLSSAIELVSHWTGVF..EKRGIP
HEMK2_HUMAN 1-214 ....
MTQ2_YEAST 1-221 ....
PrmC_HALVD 1-198 ....
PrmC_ECOLI 1-277
α2 α3 α4 α5 β1
20 30 40 50 60 70
PrmC_ECOLI 1-277 SPRRDAEILLEHVTGRGRTF..ILAFGE.TQLTDEQCQQLDALLTRRRDGEPIAHLTGVR
MTQ1_YEAST 1-314 RIRPGLHLLLPECRTL..EQAKLEYKWLTEELP..PDKSIRWACLQRYKHVPLQYILRSQ
HEMK1_HUMAN 1-338 EARESSEYIVAHVLGAKTFQSLRPALWT.QPLTSQQLQCIRELSSRRLQRMPVQYILGEW
HEMK2_HUMAN 1-214 ....MAGEN
MTQ2_YEAST 1-221 ....
PrmC_HALVD 1-198 ....
PrmC_ECOLI 1-277
TT β2 TT α6 β3
80 90 100 110 120
PrmC_ECOLI 1-277 EFWSLPL....FVSPATLIPRPDTECLVEQALARLPEQ........PCRILDLGTGTGAI
MTQ1_YEAST 1-314 PFGALDI....VCKPGVLIPRWETEEWVMAIIRALNNS.MLSRHTIPLHICDTFTGTGCI
HEMK1_HUMAN 1-338 DFQGLSL....RMVPPVFIPRPETEELVEWVLEEVAQRSHAVGSPGSPLILEVGCGSGAI
HEMK2_HUMAN 1-214 FATPFHGHVGRGAFSDVYEPAEDTFLL.LDALEAAAAELA.....GVEICLEVGSGSGVV
MTQ2_YEAST 1-221 ...MLPTPYVKCDYDKVYEPAEDSFLI.LDCLEKEHDFLKQKFGNRLAIVCEIGSGSGIV
PrmC_HALVD 1-198 ...MTDLAERRGMNTSVYQPAEDSGLL.AQAA..........VGRVSGRVLEVGTGSGWV
GxGxG type SAM binding motif
PrmC_ECOLI 1-277
α7 β4 α8 β5
130 140 150 160 170
PrmC_ECOLI 1-277 ALALASE....RPDCEIIAVDRMPDAVSLAQRNAQHLAIKNIH......ILQSDWF...S
MTQ1_YEAST 1-314 ALALSHG....IANCTFTAIDVSTRAIKLVKENMLKNKVSGGKLVQ.HNILSSKA....S
HEMK1_HUMAN 1-338 SLSLLSQ....LPQSRVIAVDKREAAISLTHENAQRLRLQDRIWIIHLDMTSERSW...T
HEMK2_HUMAN 1-214 SAFLASM.I..GPQALYMCTDINPEAACTLETARCN.KV...H...IQPVITDLVKGLL
MTQ2_YEAST 1-221 TTFLMQNKIIPQENSIHLAVDINPWALEATLDTAKLN.SCKSSF...LEVIQADLNS...
PrmC_HALVD 1-198 AEQLATK.....TDADVVASDLNPHACRQAAERAAALRADGHRG...FEVVRGSLVE...
PrmC_ECOLI 1-277
η1 β6 α9 η2 TTT α10 α11 η3
180 190 200 210 220
PrmC_ECOLI 1-277 ALAGQQFAMIVSNPPYIDEQDPHLQQGDV...RFEPLTALVAADSGMADIVHIIEQSRNA
MTQ1_YEAST 1-314 DEYPSHIDILTGNPPYIRKRDFNRDVKTS.VKLFEPRLALVGELEC......YINLVNYW
HEMK1_HUMAN 1-338 HLPWGPMDLIVSNPPYVFHQDMEQLAPEI..RSYEDPAALDGGEEGMDIITHILALAPRL
HEMK2_HUMAN 1-214 PRLTEKVDLLVFNPPYVVTP..PQEVGS.....HGIEAAWAGGRNGREVMDRFFPLVPDL
MTQ2_YEAST 1-221 SIRNNQVDVLIFNPPYVPAECVPDVPGSREEADQWLDLALLGGKDGMAITDKLLRQLEQI
PrmC_HALVD 1-198 PFRDDAFDAVAFNPPYLPEDL.......EAARDDWMEVALTGGEDGREIIDPFLDTVGRV
NPPY motif
PrmC_ECOLI 1-277
β7 α12 β8 TT β9
230 240 250 260 270
PrmC_ECOLI 1-277 LVSGGFLLLEHGWQQ.GEAVRQAFIL..AGYHD.V...ETCRDYGDNERVT.........
MTQ1_YEAST 1-314 LPKTDSFFYEIGDVEQFNYVERRIKE..DSYLSRIWSIGLKYDSNGKARVVYGFKATPKG
HEMK1_HUMAN 1-338 LKDSGSIFLEVDPRH.PELVSSWLQSRPDLYLNLV...AVRRDFCGRPRFL.........
HEMK2_HUMAN 1-214 LSPRGLFYLVTIKENNPEEILKIMKT................KGLQGTTA.........
MTQ2_YEAST 1-221 LSPDGVAYILFCARNKPKEVIKRFVDT................YKWNVKLI.........
PrmC_HALVD 1-198 LKPGGTVFLLVSSFTGYDEVLARAEA.................NGFHEVV.........
PrmC_ECOLI 1-277
PrmC_ECOLI 1-277 ...LGRYYQ...................
MTQ1_YEAST 1-314 RILHQIFASFGTIRHLATALSGHKANCN
HEMK1_HUMAN 1-338 ...HIRRSGP..................
HEMK2_HUMAN 1-214 ...LSRQAGQETLSVLKFTKS.......
MTQ2_YEAST 1-221 ...ETRKAGWEVLSVYSFTR........
PrmC_HALVD 1-198 ...VQESYPYETLTVLALE.........

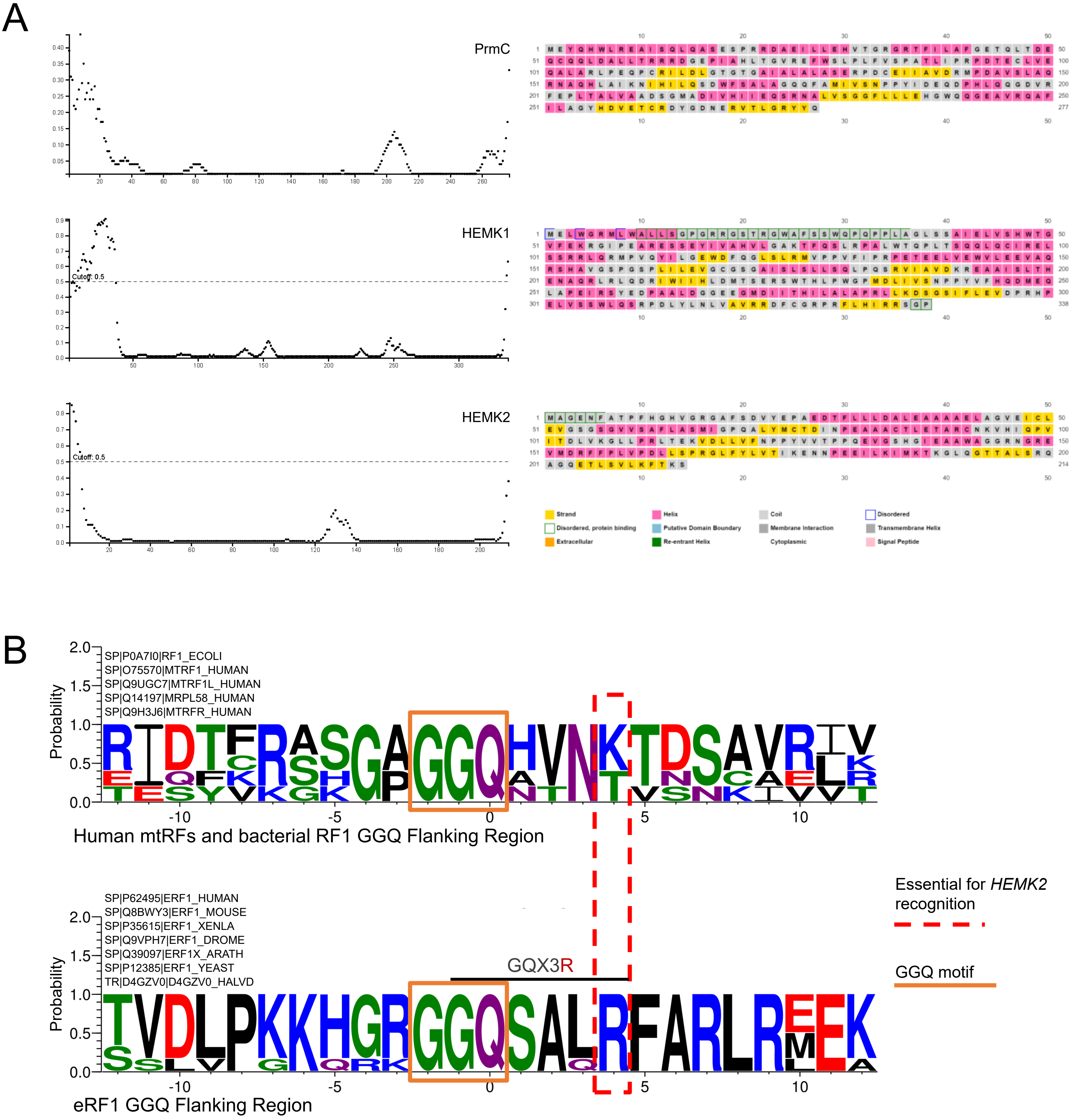
A
PrmC
HEMK1
Cutoff: 0.5
HEMK2
Cutoff: 0.5
Strand
Helix
Coil
Disordered
Disordered, protein binding
Putative Domain Boundary
Membrane Interaction
Transmembrane Helix
Extracellular
Re-entrant Helix
Cytoplasmic
Signal Peptide
B
SP|P0A7I0|RF1_ECOLI
SP|O75570|MTRF1_HUMAN
SP|Q9UGC7|MTRF1L_HUMAN
SP|Q14197|MRPL58_HUMAN
SP|Q9H3J6|MTRFR_HUMAN
Probability
Human mtRFs and bacterial RF1 GGQ Flanking Region
SP|P62495|ERF1_HUMAN
SP|Q8BWY3|ERF1_MOUSE
SP|P35615|ERF1_XENLA
SP|Q9VPH7|ERF1_DROME
SP|Q39097|ERF1X_ARATH
SP|P12385|ERF1_YEAST
TR|D4GZV0|D4GZV0_HALVD
GQX3R
Probability
eRF1 GGQ Flanking Region
Essential for HEMK2 recognition
GGQ motif

Fig_S3 Fang et al.

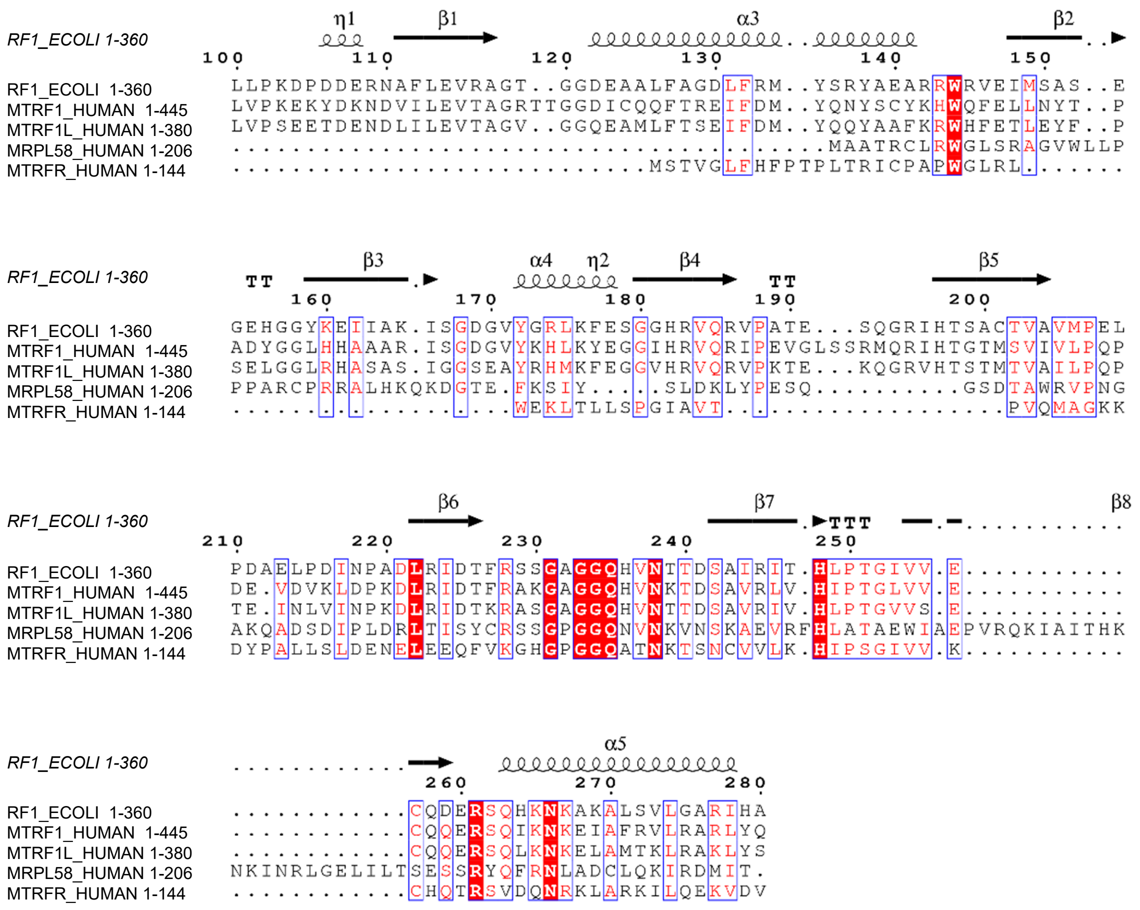

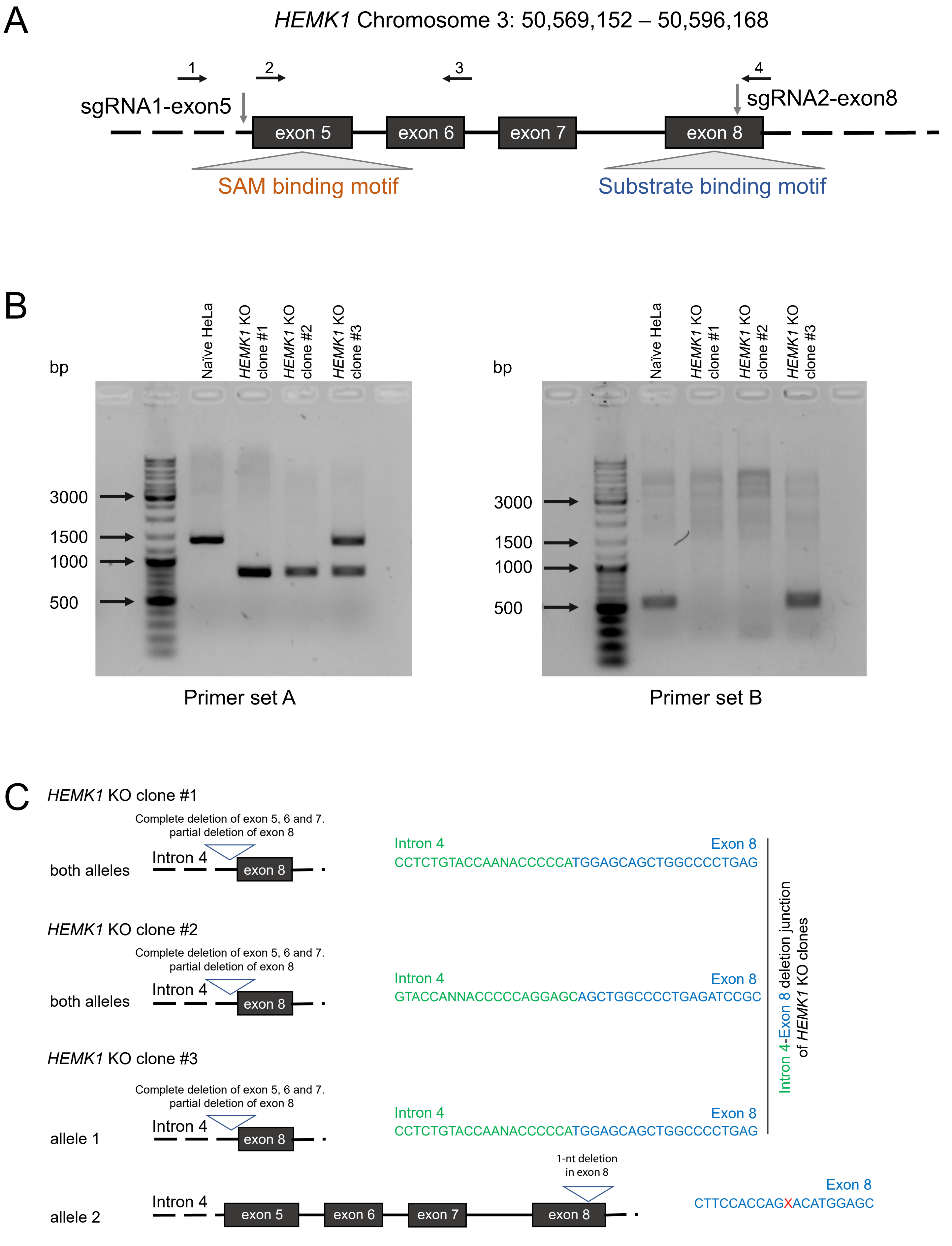
A
HEMK1 Chromosome 3: 50,569,152 – 50,596,168
1
2
3
4
sgRNA1-exon5
sgRNA2-exon8
exon 5
exon 6
exon 7
exon 8
SAM binding motif
Substrate binding motif
B
bp
Naïve HeLa
HEMK1 KO clone #1
HEMK1 KO clone #2
HEMK1 KO clone #3
3000
1500
1000
500
Primer set A
Primer set B
C
HEMK1 KO clone #1
Complete deletion of exon 5, 6 and 7. partial deletion of exon 8
both alleles
Intron 4
exon 8
Intron 4
Exon 8
CCTCTGTACCAANACCCCCATGGAGCAGCTGGCCCCTGAG
HEMK1 KO clone #2
Complete deletion of exon 5, 6 and 7. partial deletion of exon 8
both alleles
Intron 4
exon 8
Intron 4
Exon 8
GTACCANNACCCCCAGGAGCAGCTGGCCCCTGAGATCCGC
HEMK1 KO clone #3
Complete deletion of exon 5, 6 and 7. partial deletion of exon 8
allele 1
Intron 4
exon 8
Intron 4
Exon 8
CCTCTGTACCAANACCCCCATGGAGCAGCTGGCCCCTGAG
Intron 4-Exon 8 deletion junction of HEMK1 KO clones
1-nt deletion in exon 8
allele 2
Intron 4
exon 5
exon 6
exon 7
exon 8
Exon 8
CTTCCACCAGXACATGGAGC

A

Cells	mtRFs OE	HEMK1 OE	Unmodified peptide	Modified peptide	% methylation
Naïve HeLa	MTRF1L	-	2018861766	24759659	1.21
HEMK1 KO clone #1	MTRF1L	-	1338842996	0	0
HEMK1 KO clone #1	MTRF1L	WT	216982198	42771708	20.48
HEMK1 KO clone #1	MTRF1L	$HEMK1^{Y242E}$	35557205	0	0
Naïve HeLa	MTRF1	-	241224868	4329953	1.76
HEMK1 KO clone #1	MTRF1	-	100367781	0	0
HEMK1 KO clone #1	MTRF1	WT	216982198	42771708	16.46
Naïve HeLa	MRPL58	-	2157227983	3831870	0.18
HEMK1 KO clone #1	MRPL58	-	1450827245	0	0
HEMK1 KO clone #1	MRPL58	WT	2201046389	189016672	7.91
Naïve HeLa	MTRFR	-	55652312	828716	1.52
HEMK1 KO clone #1	MTRFR	-	47624452	0	0
HEMK1 KO clone #1	MTRFR	WT	57079627	54050476	48.63

B

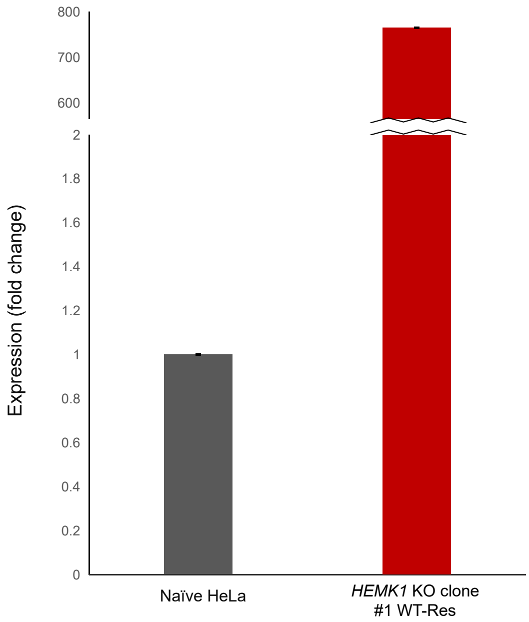

C

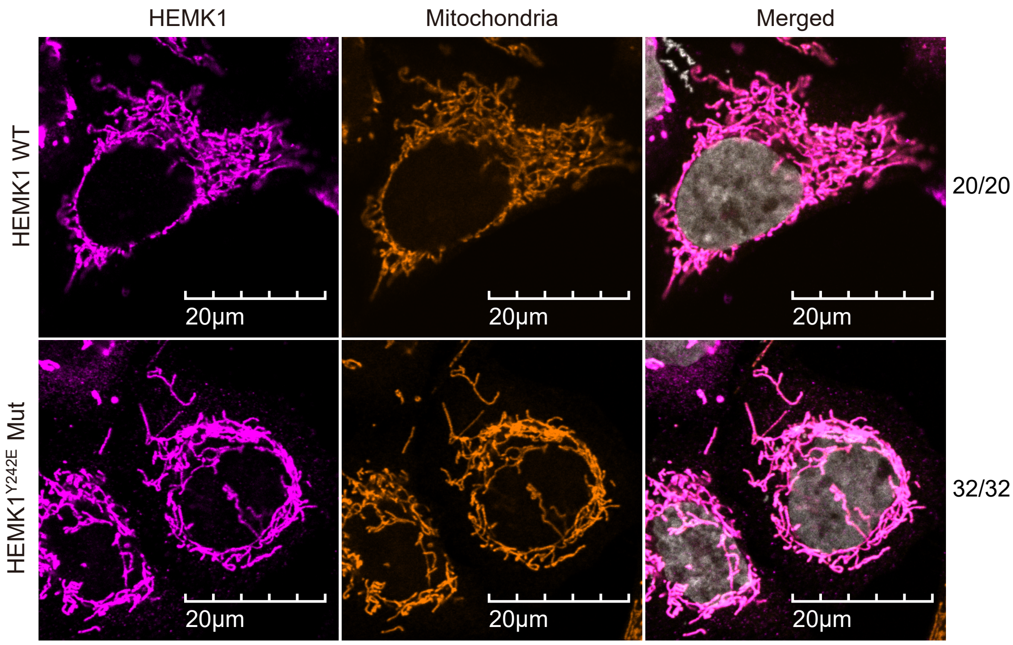

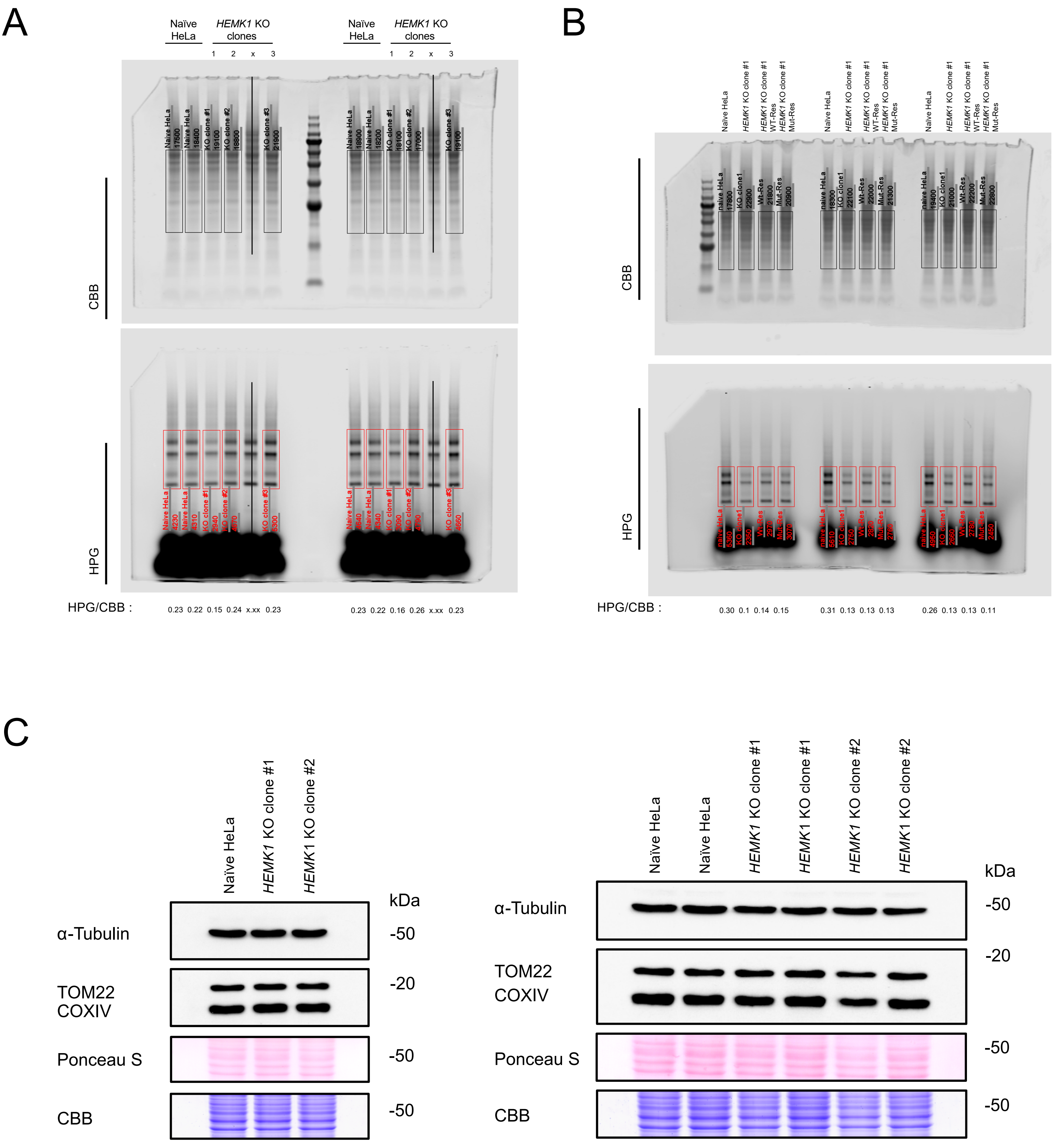
A
Naïve HeLa
HEMK1 KO clones
1 2 x 3
CBB
HPG
HPG/CBB :
0.23 0.22 0.15 0.24 x.xx 0.23
0.23 0.22 0.16 0.26 x.xx 0.23
B
Naïve HeLa
HEMK1 KO clone #1
HEMK1 KO clone #1 WT-Res
HEMK1 KO clone #1 Mut-Res
CBB
HPG
HPG/CBB :
0.30 0.1 0.14 0.15
0.31 0.13 0.13 0.13
0.26 0.13 0.13 0.11
C
Naïve HeLa
HEMK1 KO clone #1
HEMK1 KO clone #2
kDa
α-Tubulin
TOM22
COXIV
Ponceau S
CBB
-50
-20
-50
-50
Naïve HeLa
Naïve HeLa
HEMK1 KO clone #1
HEMK1 KO clone #1
HEMK1 KO clone #2
HEMK1 KO clone #2
kDa
α-Tubulin
TOM22
COXIV
Ponceau S
CBB
-50
-20
-50
-50

A

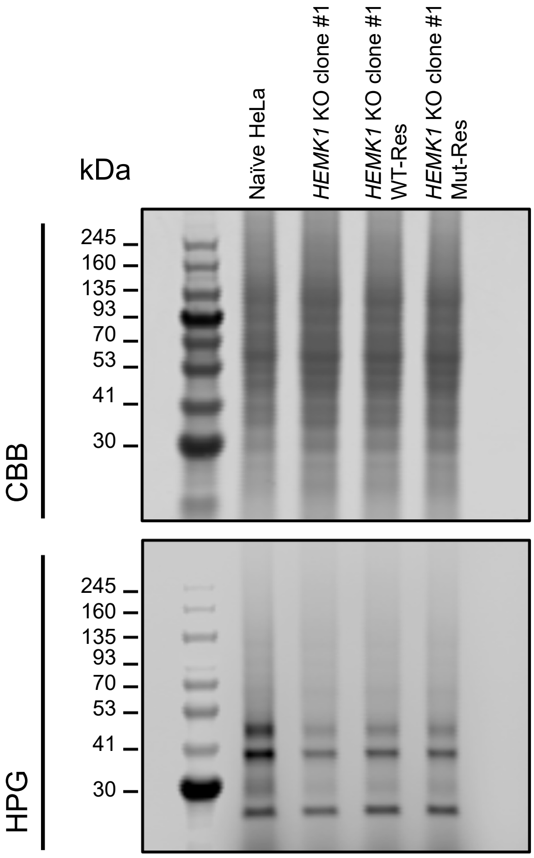
kDa
Naïve HeLa
HEMK1 KO clone #1
HEMK1 KO clone #1 WT-Res
HEMK1 KO clone #1 Mut-Res
245
160
135
93
70
53
41
30
CBB
HPG

B

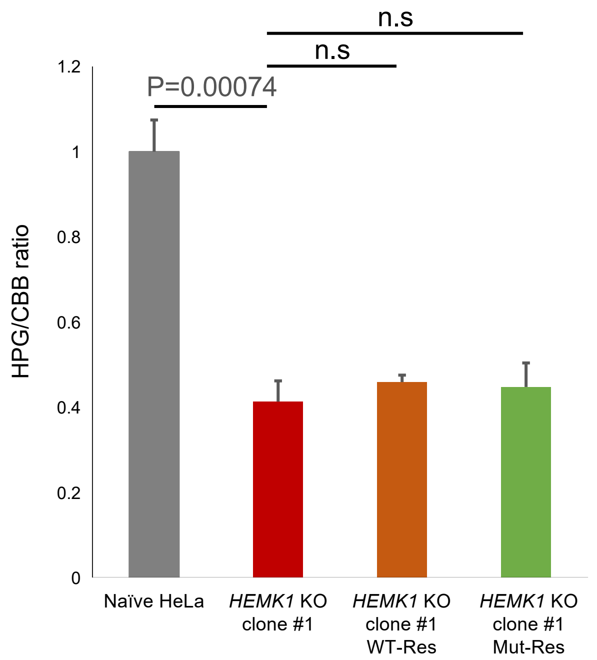
HPG/CBB ratio
1.2
1
0.8
0.6
0.4
0.2
0
P=0.00074
n.s
n.s
Naïve HeLa
HEMK1 KO clone #1
HEMK1 KO clone #1 WT-Res
HEMK1 KO clone #1 Mut-Res

C

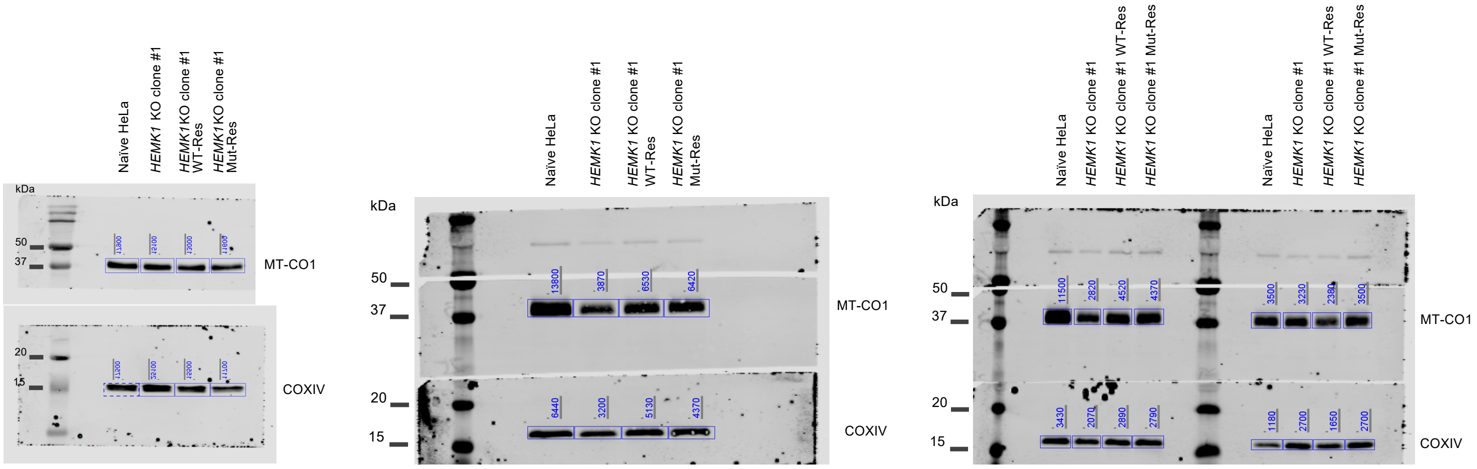
Naïve HeLa
HEMK1 KO clone #1
HEMK1KO clone #1 WT-Res
HEMK1KO clone #1 Mut-Res
HEMK1 KO clone #1 WT-Res
HEMK1 KO clone #1 Mut-Res
kDa
50
37
20
15
MT-CO1
COXIV

D

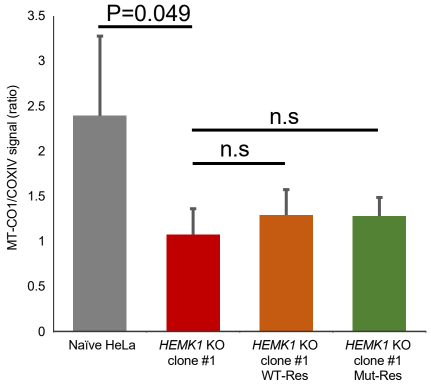
MT-CO1/COXIV signal (ratio)
3.5
3
2.5
2
1.5
1
0.5
0
P=0.049
n.s
n.s
Naïve HeLa
HEMK1 KO clone #1
HEMK1 KO clone #1 WT-Res
HEMK1 KO clone #1 Mut-Res

A

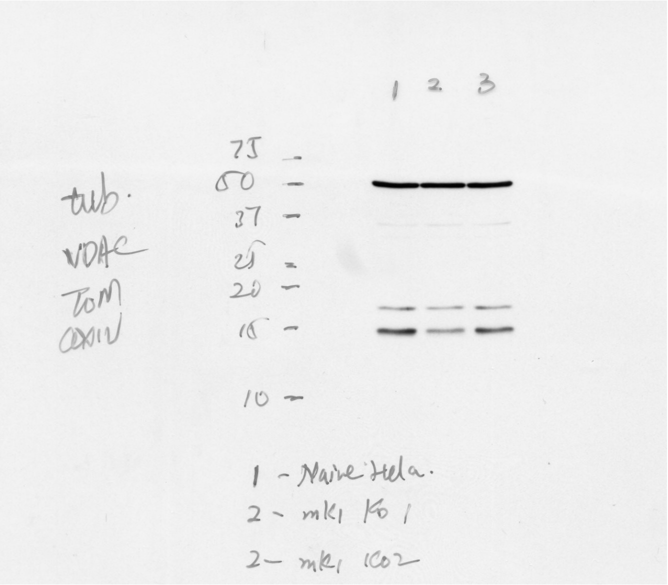
1 2 3
75
50
37
25
20
15
10
tub.
VDAC
TOM
1 – Naive Hela.
2 – mk1 ko1
2 – mk1 ko2

B

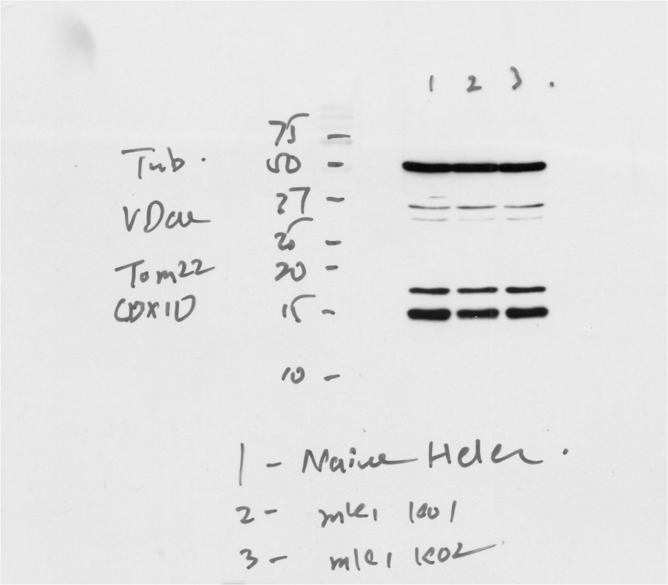
1 2 3
75
50
37
25
20
15
10
Tub.
VDac
Tom22
COXIV
1 – Naive Hela.
2 – mk1 ko1
3 – mk1 ko2

C

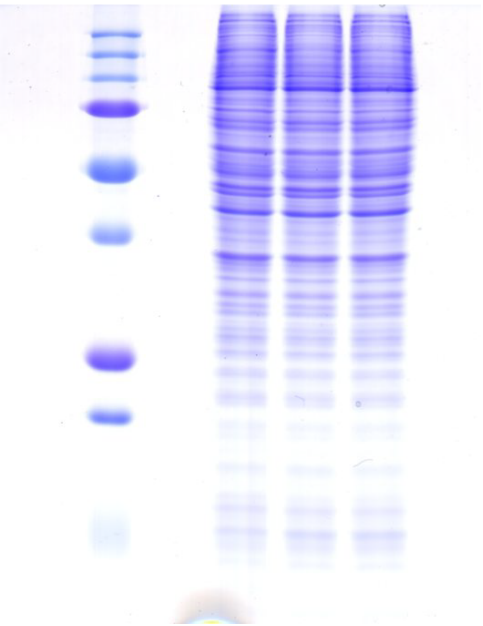

D

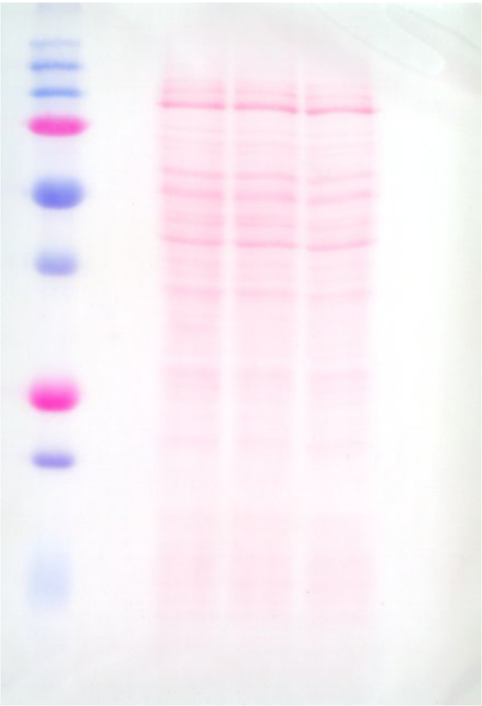

Fig_S9 Fang et al.

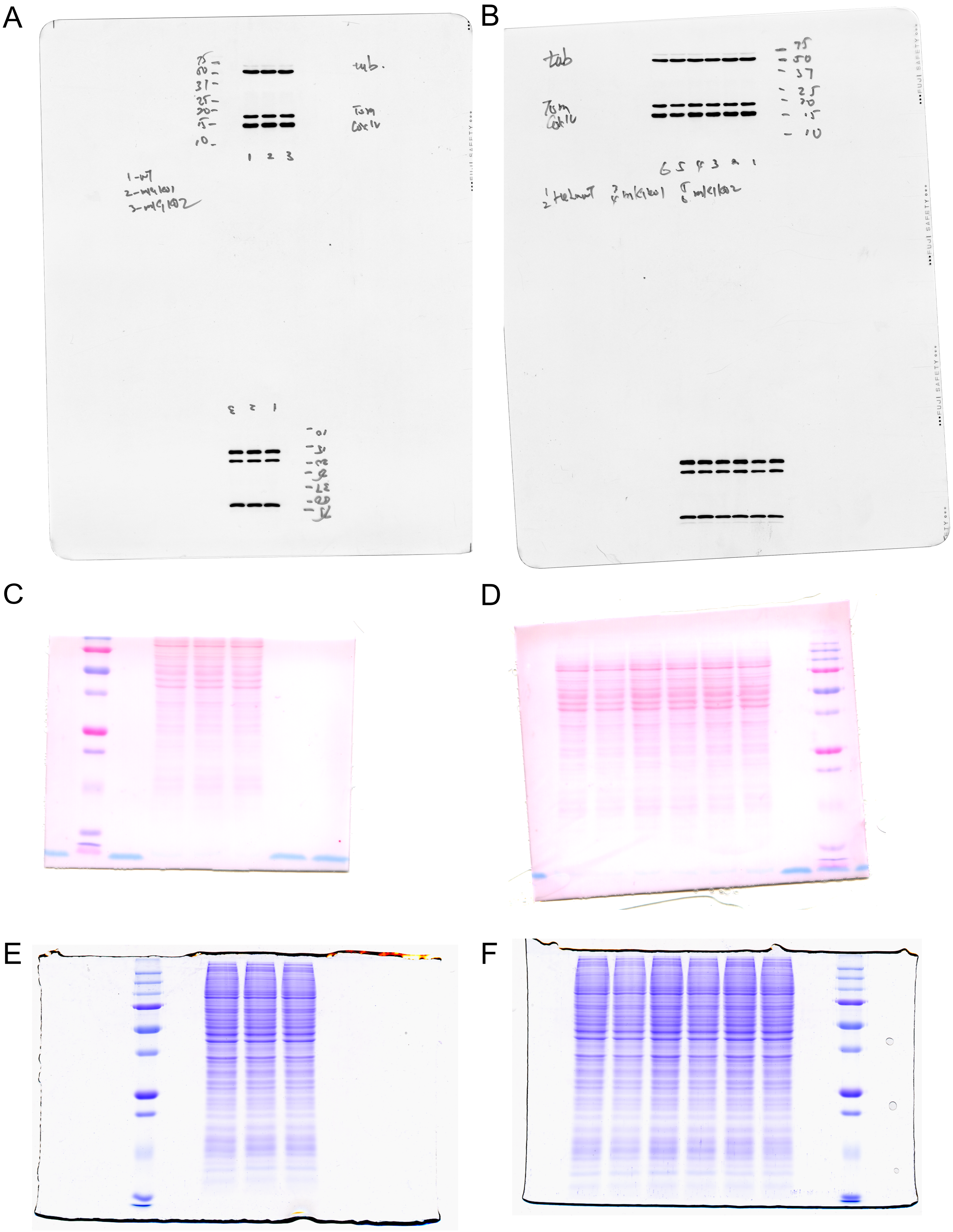

A

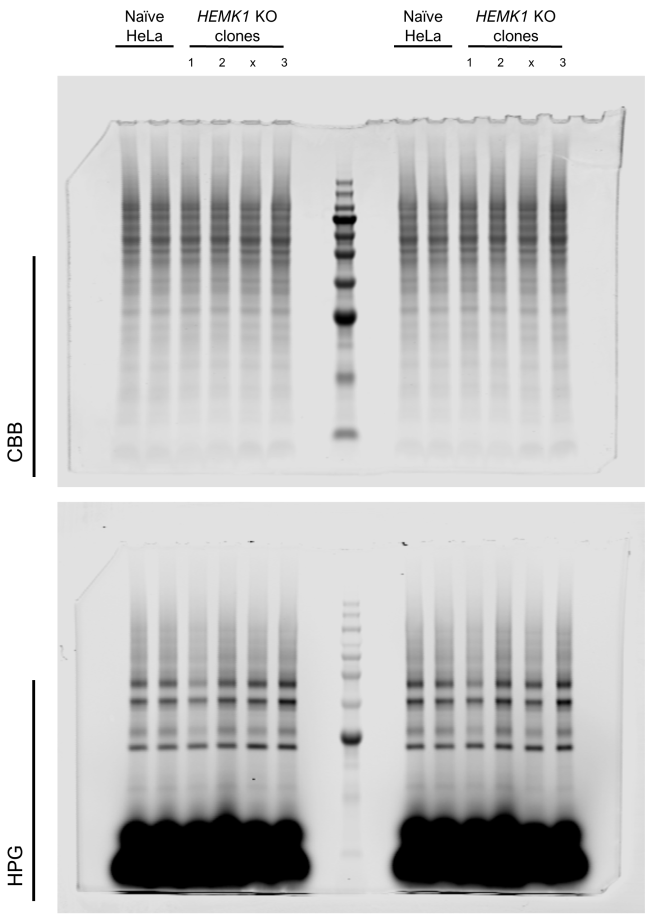
Naïve HeLa
HEMK1 KO clones
1
2
x
3
Naïve HeLa
HEMK1 KO clones
1
2
x
3
CBB
HPG

B

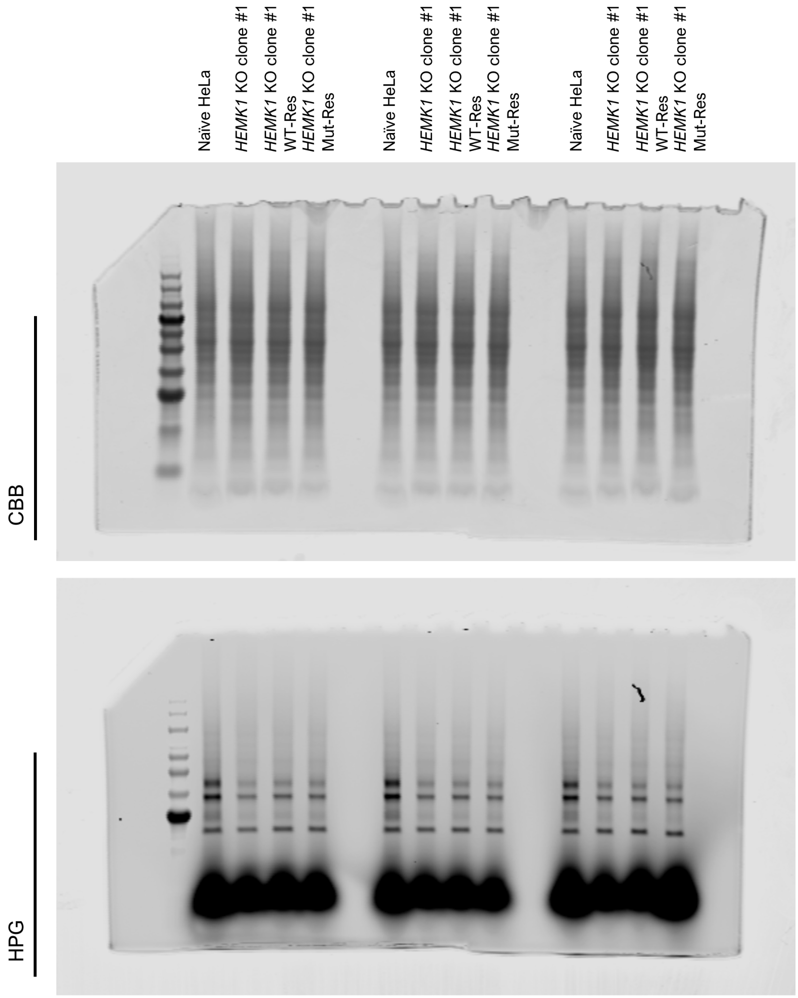
Naïve HeLa
HEMK1 KO clone #1
HEMK1 KO clone #1 WT-Res
HEMK1 KO clone #1 Mut-Res
Naïve HeLa
HEMK1 KO clone #1
HEMK1 KO clone #1 WT-Res
HEMK1 KO clone #1 Mut-Res
Naïve HeLa
HEMK1 KO clone #1
HEMK1 KO clone #1 WT-Res
HEMK1 KO clone #1 Mut-Res
CBB
HPG

A

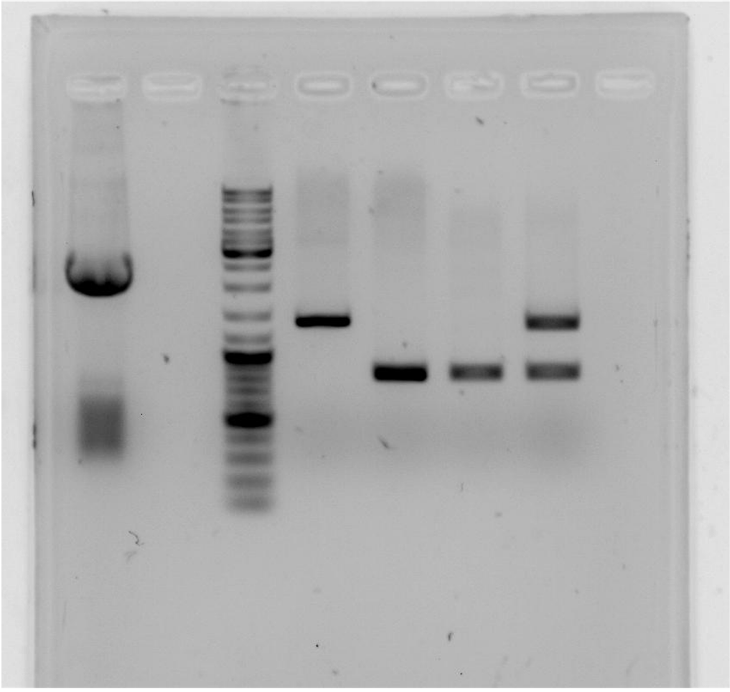

* The leftmost lane is a plasmid unrelated to this manuscript

B

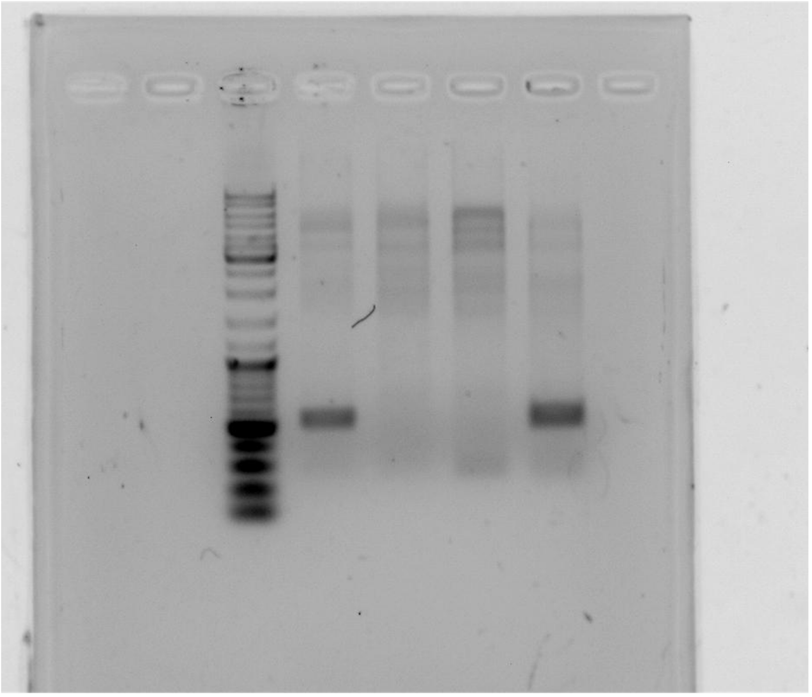

Supplement: Supplementary file 1 — Supplementary Information 1. [file 41598_2022_8061_MOESM1_ESM.pdf]
